# Palmitic Acid Inhibits the Proliferation and Hypertrophy of Antler Chondrocytes by Disrupting Mitochondria-Associated Endoplasmic Reticulum Membrane Function

**DOI:** 10.3390/antiox15091108

**Published:** 2026-09-02

**Authors:** Qiaoling Zhang, Zhanqing Yang, Xueyuan Yao, Yinfei Xing, Chenhao Wang, Baiyu Li, Zhanpeng Yue, Bin Guo

**Affiliations:** College of Veterinary Medicine, Jilin University, Changchun 130062, China

**Keywords:** palmitic acid, antler chondrocyte, NOTCH1 signaling, MAM, mitochondrion

## Abstract

As the only mammalian appendage capable of complete regeneration, deer antlers serve as an invaluable model to investigate cartilage regrowth, but the underlying mechanism remains unclear. This study revealed that addition of palmitic acid (PA), an abundant long-chain saturated free fatty acid, inhibited the proliferation and hypertrophy of antler chondrocytes while promoting chondrocyte apoptosis. PA treatment activated NOTCH1 signaling and restrained the transport of Ca^2+^ from the cytosol to the endoplasmic reticulum (ER) via RBPJ (recombination signal-binding protein for immunoglobulin kappa J region)-targeted TMTC4 (transmembrane O-mannosyltransferase targeting cadherins 4), resulting in a reduction in ER Ca^2+^. Meanwhile, PA disrupted the structure and function of mitochondria-associated ER membranes (MAMs) via TGM2 (transglutaminase 2) through the cytosolic Ca^2+^-mediated PPP3CB (protein phosphatase 3 catalytic subunit beta)-NFATC2 (nuclear factor of activated T cells cytoplasmic 2) pathway. Further analysis demonstrated that PA induced mitochondrial dysfunction via MAM-mediated mitochondrial Ca^2+^ insufficiency, thereby restricting mitophagy and attenuating lysosomal acidification. This caused the leakage of mitochondrial reactive oxygen species (mtROS) from depolarized mitochondria into the cytosol via the mitochondrial permeability transition pore, thereby inducing lipid peroxidation, while the addition of ROS scavengers prevented the negative effects of chondrocyte proliferation and hypertrophy and protected chondrocytes from apoptosis in the context of PA. Collectively, PA treatment regulated the proliferation, apoptosis and hypertrophy of antler chondrocytes by disrupting MAM function.

## 1. Introduction

Antlers are bony cranial appendages that grow from the foreheads of male deer and possess various pharmacological properties that are valuable in traditional Chinese medicine [1,2]. Antlers are the only mammalian organs that undergo annual cycles of complete regeneration, accompanied by an extraordinary growth rate of up to 2.75 cm per day [2,3]. Antlers regrow from the pedicle, where mesenchymal cells and chondrocytes undergo rapid proliferation and differentiation and form vascularized cartilage and bone, thus providing an unparalleled model to explore limb regeneration, angiogenesis, chondrogenesis, endochondral ossification, and cartilage and bone development [1,2].

Free fatty acids (FFAs), key energy substrates in organisms, undergo catabolism via β-oxidation and are grouped into short-, medium-, and long-chain species according to their aliphatic tail length [4,5]. In addition, FFAs also serve as signaling molecules that modulate various physiological and pathological processes through interactions with membranes or intracellular proteins [4,5]. Extensive evidence has established the roles of short- and medium-chain FFAs in skeletal health [6,7,8]. Long-chain FFAs are further categorized as polyunsaturated, monounsaturated or saturated FFAs [8]. Both polyunsaturated FFAs and monounsaturated FFAs have been reported to participate in bone and cartilage development, and their supplementation can prevent cartilage destruction, enhance osteoblast activity and alleviate the phenotypes of osteoporosis, osteoarthritis and rheumatoid arthritis [8,9,10]. In contrast, saturated FFAs, particularly palmitic acid (PA), the most abundant long-chain saturated FFA, account for 28–32% of total serum FFAs and can exhibit toxic effects on articular chondrocytes, where they can trigger oxidative stress, mitochondrial dysfunction, and apoptosis, resulting in cartilage degeneration [11,12]. Furthermore, PA can elicit endoplasmic reticulum (ER) stress and activate the unfolded protein response signaling pathway in chondrocytes, thereby promoting cartilage lesion formation in vivo [13,14]. Notably, oleic acid, a monounsaturated fatty acid, can partially reverse or prevent PA-induced mitochondrial dysfunction [12]. Meanwhile, studies have found that PA levels decline in soft (unossified) antler compared with the pedicle and increase in ossified antler, implying the potential involvement of PA in antler regeneration [15,16]. Nevertheless, it remains largely unknown whether PA can directly modulate cartilage development in dynamic antler regeneration.

The mitochondria-associated ER membrane (MAM) is a contact site between the ER and mitochondria, with a distance of approximately 10–30 nm. It serves as a crucial platform for calcium ion (Ca^2+^) and lipid exchange [17]. Accumulating data reveal that MAMs play an important role in various biological functions, such as steroidogenesis, insulin secretion, glucose homeostasis, metabolism, inflammatory responses, mitochondrial oxidative phosphorylation, angiogenesis, etc. [18,19,20,21]. However, it is uncertain whether MAMs are involved in cartilage development. Moreover, little information is available regarding the regulation of PA on MAMs.

Mitophagy, a regulatory mechanism for eliminating damaged mitochondria, can protect chondrocytes from degeneration. [22,23]. Defective mitophagy leads to dysfunctional mitochondria accumulation, persistent ROS generation, and cartilage degradation [24,25]. Given that PA elicits mitochondrial dysfunction, characterized by elevated superoxide, reduced respiratory capacity, and decreased ATP production, PA may interfere with mitophagy [11,12]. However, whether PA affects mitophagy or whether MAM dysfunction contributes to PA-induced mitochondrial oxidative stress and impaired mitophagy remains unexplored.

The present study reveals that PA inhibits the hypertrophy and proliferation of antler chondrocytes and facilitates chondrocyte apoptosis. After activation of NOTCH1 signaling, PA prevented the transport of cytosolic Ca^2+^ into the ER via recombination signal-binding protein for immunoglobulin kappa J region (RBPJ)-targeted TMTC4 (transmembrane O-mannosyltransferase targeting cadherins 4), resulting in the reduction of ER Ca^2+^ and accumulation of cytosolic Ca^2+^. The latter disrupted the structure and function of MAMs via Ca^2+^-mediated transglutaminase 2 (TGM2) and then induced mitochondrial dysfunction. This was followed by impairment of mitophagy and leakage of mitochondrial reactive oxygen species (mtROS), ultimately leading to lipid peroxidation, defective hypertrophy and proliferation, and increased apoptosis in antler chondrocytes.

## 2. Materials and Methods

### 2.1. Antler Collection and Chondrocyte Treatment

Antler tissues were collected from the growing tips (distal 5 cm) of five healthy three-year-old sika deer. The chondrocytes were isolated as reported previously [26]. The primary antler chondrocytes were characterized as previously described [27] and then cryopreserved in liquid nitrogen for subsequent experiments. All animal experiment procedures were approved by the Committee for the Ethics on Animal Care and Use of Jilin University (SY201905030).

Passage-3 antler chondrocytes were cultured in complete medium consisting of 90% DMEM-High Glucose and 10% fetal bovine serum. After saponification with NaOH (0.1 M) for 30 min and subsequent mixture with 20% BSA solution at 1:4, PA (100 μM, final PA:BSA molar ratio of approximately 3.3:1) was administrated into the antler chondrocytes. Then, the chondrocytes were treated with the γ-secretase inhibitor DAPT (10 μM, Selleck, Houston, TX, USA), RBPJ inhibitor RIN1 (2 μM, MCE, Monmouth Junction, NJ, USA), intracellular Ca^2+^ chelator BAPTA-AM (BAPTA, 10 μM, Selleck), calcineurin inhibitor Tacrolimus (FK506, 50 nM, MCE), nuclear factor of activated T cell (NFATC) inhibitor VIVIT peptide (VIVIT, 2 μM, MCE), mitochondrial permeability transition pore (mPTP) opening inhibitor ER000444793 (ER093, 2 μM, MCE), ROS scavenger N-acetylcysteine (NAC, 1 mM, Beyotime, Shanghai, China) or mitochondria-targeted ROS scavenger Mito-TEMPO (MT, 1, 5 or 10 μM, MCE) prior to the addition of PA. The control received the vehicle only.

### 2.2. In Situ Hybridization

In situ hybridization was performed as previously described [28]. Briefly, NOTCH1 (116 bp) and RBPJ (157 bp) fragments were amplified using specific primers and then cloned into pGEM-T plasmid (Promega, Madison, WI, USA). These plasmids were further amplified with T7 and SP6 primers to obtain the templates for probe labeling. Digoxigenin (DIG)-labeled antisense and sense cRNA probes were generated in vitro using a DIG RNA Labeling Kit (Roche, Mannheim, Germany). Frozen sections (10 μm thickness) were prepared and then fixed with 4% paraformaldehyde for 1 h. After incubation with DIG-labeled probes for 20 h, sheep anti-DIG antibody conjugated to alkaline phosphatase was administered to the cryosections. Signals were visualized using BCIP/NBT and then stained with methyl green (Sigma, St. Louis, MO, USA). The primers for cDNA probe synthesis were as follows: NOTCH1 (5′-AACCGTAGCTCCTGAGAGCA-3′ and 5′-AGAGTCTGATCGTGCCCACT-3′) and RBPJ (5′-GAGCGAGGGGATCAAACAGT-3′ and 5′-CAGAACAACCATCGCGTTCC-3′).

### 2.3. Construction and Transfection of Overexpression Plasmid

Full-length TGM2 cDNA fragment was amplified by the specific primers designed by SnapGene 7.0.2 software according to the *Cervus elaphus* TGM2 mRNA sequence (accession number XM_043883725). The following primer sequences were used: 5′-CTGAGATCTATGGCCGAGGAGCTGGTCCTG; and 5′-CTGGTCGACTTAGGAGGGGCCAACGATGAC. After restriction enzyme digestion, the fragment was inserted into the pCMV-4 × Flag vector. Subsequently, TGM2 overexpression plasmid was transfected into antler chondrocytes using Lipofectamine 3000 reagent (Invitrogen, Thermo Fisher Scientific, Waltham, MA, USA), followed by the addition of PA.

### 2.4. RNA Interference

Small interfering RNAs (siRNAs) targeting TMTC4, NOTCH1, PPP3CB or NFATC2 were designed via the DSIR web tool (http://biodev.cea.fr/DSIR/, accessed on 17 January 2026) according to the corresponding mRNA sequences from *Cervus elaphus*, and then synthesized by GenePharma (Suzhou, China). After the introduction of these siRNAs, antler chondrocytes were treated with PA or RIN1 plus PA. Scrambled siRNA served as a negative control (NC). The siRNA sequence and accession numbers are provided in Table S1.

### 2.5. Transcriptome Sequencing (RNA-Seq)

After treatment with RBPJ inhibitor RIN1, total RNA was extracted using TRIzol reagent (Life technologies, Carlsbad, CA, USA) according to the manufacturer’s instructions. RNA concentration and purity were determined via NanoDrop 2000 spectrophotometer (Thermo Fisher Scientific, Waltham, MA, USA), while RNA integrity was evaluated using the Agilent Bioanalyzer 2100 system (Agilent Technologies, Santa Clara, CA, USA) (RIN values for all samples were >9.0). A total amount of 1 μg RNA per sample (*n* = 3 biological replicates per group) was utilized to construct the libraries using Hieff NGS Ultima Dual-mode mRNA Library Prep Kit (Yeasen, Shanghai, China). Libraries were sequenced on an Illumina NovaSeq6000 platform at Biomarker Technologies Co., Ltd (Beijing, China). Clean reads were mapped to the *Cervus elaphus* genome after raw data processing. Differentially expressed genes (DEGs) were screened with the criteria for *p*-value < 0.05 and |log2(fold change, FC)| ≥ 1.5 and then used to perform KEGG and GO analysis using the BMKCloud platform.

### 2.6. Real-Time PCR

After various treatments, total RNA was extracted from antler chondrocytes using TRlzol reagent, and then reversely transcribed into cDNA using the All-In-One 5 × RT MasterMix (Applied Biological Materials Inc., Richmond, BC, Canada). Real-time PCR was performed using the corresponding primers (Table S2) on Applied Biosystems QuantStudio 1 (Thermo Fisher Scientific) as described previously [26]. The reaction conditions were as follows: 95 °C for 3 min, followed by 40 cycles of 95 °C for 15 s and 60 °C for 1 min. Finally, the expression levels of different genes were analyzed using the 2^−ΔΔCt^ method together with normalization to *GAPDH*.

### 2.7. Western Blotting

Proteins were extracted from antler chondrocytes using RIPA Lysis Buffer (Beyotime). Equal proteins were used to perform SDS-PAGE and then transferred to PVDF membranes. After probing with various antibodies (Table S3), membranes were incubated with HRP-conjugated secondary antibody (Proteintech, Rosemont, IL, USA) and visualized using ECL chemiluminescent substrates.

### 2.8. Cell Apoptosis

Antler chondrocytes were incubated with FITC-labeled Annexin V and propidium iodide (Beyotime) for 20 min after various treatments and then analyzed using flow cytometry (Beckman Coulter, Brea, CA, USA) or visualized under a fluorescence microscope (Olympus, Tokyo, Japan). Simultaneously, caspase 3 (CASP3) activity was assessed using the corresponding kit (Beyotime).

### 2.9. Cell Proliferation

Chondrocyte proliferation was assessed using the EdU Cell Proliferation Kit with Alexa Fluor 488 (Beyotime). Briefly, after incubation with EdU (10 μM) for 2 h, antler chondrocytes were fixed with 4% paraformaldehyde and then permeabilized with 0.3% Triton-X100, following by click reaction solution treatment (including Azide 488). Finally, chondrocytes were analyzed using flow cytometry or visualized under fluorescence microscope after the nuclear staining of Hoechst 33342 (Hoechst).

### 2.10. Cell Viability Analysis

Cell viability was evaluated using an LDH release assay (Beyotime). After various treatments, culture supernatants were collected and then incubated with LDH detection working solution for 30 min. Absorbance was measured at 490 nm. Cells treated with LDH release reagent served as the maximum release control, while untreated cells served as the negative control. Cell viability was calculated according to the operating instructions of the manufacturer.

### 2.11. Transmission Electron Microscopy (TEM)

After transfection with TGM2 overexpression plasmid and addition of PA, antler chondrocytes were fixed with 2.5% glutaraldehyde and 1% osmic acid and then embedded into epoxy resin. Sections (60 nm thickness) were stained with uranyl acetate and lead citrate, followed by visualization using a HITACHI HT7800 transmission electron microscope operated at an accelerating voltage of 80 kV. Finally, the distance between ER and mitochondria was measured using ImageJ software (version 1.53c).

### 2.12. Analysis of MAMs

After the introduction of TGM2 overexpression plasmid and addition of PA, antler chondrocytes were incubated with ER-Tracker Red (1 μM, Beyotime) and Mito-Tracker Green (20 nM, Beyotime) fluorescent probes for 30 min. Images were captured using a confocal laser scanning microscope (Nikon Corporation, Tokyo, Japan) and applied to perform colocalization analysis with Pearson’s correlation coefficient (PCC) using ImageJ software. Concurrently, the TGM2 overexpression vector was co-transfected with the SPLICS Mt-ER Long P2A plasmid (Addgene, Watertown, MA, USA; #164107), which was extensively used to detect variations in MAMs [29]. Following PA supplementation, images were obtained under confocal microscopy, and fluorescence intensity was analyzed using ImageJ software.

### 2.13. Measure of Mitochondrial Ca^2+^

After the introduction of TGM2 overexpression and the mito-GEM-GECO1 plasmid (Addgene, #32461), antler chondrocytes were treated with PA for 12 h and then cultured in Ca^2+^-free HBSS in the absence or presence of histamine (100 μM, MCE) or ATP (500 μM, Beyotime), which might stimulate Ca^2+^ transfer from the ER to mitochondria via MAMs [30,31]. Images were sequentially captured for 400 s at an interval of 20 s using a confocal microscope equipped with a ×20 objective at 450 nm (blue) and 510 nm (green) emission wavelengths and 405 nm excitation. Background-subtracted blue and green fluorescence intensities were analyzed using ImageJ software. Finally, the blue/green fluorescence ratio was calculated. Meanwhile, chondrocytes were incubated with Rhod-2 AM (5 μM, Santa Cruz) and subsequently visualized after Hoechst staining under a fluorescence microscope, followed by calculation of fluorescence intensity using ImageJ software.

### 2.14. Sequence Alignment and Molecular Docking

NOTCH1 extracellular domain sequences from *Homo sapiens* and *Cervus elaphus* were compared using DNAMAN software (version 9.0.1.116). The crystal structures of epidermal growth factor (EGF)-like 11-13 (PDB: 4D0E) and NRR (PDB: 3ETO) of NOTCH1 extracellular domain were obtained from the RCSB Protein Data Bank. The structures were used to remove the water, ligands and ions using PyMOL software (version 2.4.0), and then polar hydrogens were added by AutoDock4 software. The grid box covered the entire crystal structure. A PA 3D structure (PubChem CID: 985) was acquired and then applied to perform molecular docking. Docking was performed 10 times per independent run, with 5 independent runs conducted. For each docking pose, the binding sites of PA on the NRR were determined, and the number of bonds formed within each subdomain of the NRR (LNR-A, LNR-B, LNR-C, and HD) was counted. The average number of PA binding sites within each NRR subdomain was calculated across the 5 independent runs. Images were visualized by PyMOL, AutoDock4 or Ligplot software. Finally, amino acid residues of different binding domains in NOTCH1 extracellular domain were counted using DiscoveryStudio software (version 23.1.100.23209).

### 2.15. Analysis of Binding Between PA and NOTCH1

After antler chondrocytes were lysed, the protein concentrations in supernatants were measured. Subsequently, a protein sample (10 μg) was added to a 96-well plate that had been coated with NOTCH1 polyclonal antibody (1:1000, Proteintech) and blocked with 1% BSA. Following incubation with the BODIPY™-FL-labeled PA (50, 100 or 200 μM, Invitrogen) or vehicle for 2 h, fluorescence intensity was measured using a Multi-Detection Microplate Reader. Meanwhile, recombinant NOTCH1 NRR protein was coated on the microplate surface. After incubation with BODIPY™ FL-labeled PA, fluorescence intensity was measured. Finally, results were analyzed as F/F0, where F0 and F denote the background-subtracted fluorescence intensities from the vehicle and BODIPY™-FL-labeled PA treatment group, respectively.

### 2.16. KEGG and GO Analysis

Highly expressed genes (HEGs) of antler cartilage layers were obtained from the NCBI database (accession ID: PRJNA831044) [3]. KEGG and GO analyses were performed using the ClusterProfiler package. Images were plotted via the Bioinformatics online platform at https://www.bioinformatics.com.cn/en (accessed on 30 March 2025).

### 2.17. Analysis of Cytosolic Ca^2+^

After various treatments, antler chondrocytes were incubated with Fluo-3 AM (2.5 μM, Beyotime) for 30 min and then used to perform flow cytometry analysis to determine the cytosolic Ca^2+^ changes.

### 2.18. Measurement of ER Ca^2+^

After TMTC4 siRNA was co-transfected with pcDNA-D1ER plasmid (Addgene, #36325), an ER-targeted fluorescence resonance energy transfer (FRET) probe, antler chondrocytes were treated with PA for 12 h. Following replacement of the complete medium with DMEM containing 2-Aminoethyl Diphenylborinate (2-APB, 50 μM, Selleck) and azumolene (AZU, 20 μM, Selleck), which inhibited ER Ca^2+^ release, chondrocytes were visualized under a confocal microscope equipped with a ×100 oil immersion objective at a 435 nm excitation wavelength and 460 nm and 535 nm emission wavelengths. Meanwhile, images were consecutively captured for 400 s at intervals of 20 s using a confocal microscope with a ×20 objective to record the dynamic changes in ER Ca^2+^. Finally, images were pseudo-colored according to the emissions at 460 nm (red) and 535 nm (green) and then the green/red fluorescence ratio was analyzed using ImageJ software. ER Ca^2+^ oscillation was calculated using the F/F0, where F represents the green/red fluorescence ratio, and F0 represents the baseline fluorescence intensity at 0 s. All data are presented as relative changes in ER Ca^2+^ levels.

### 2.19. Measurement of ATP Content

After various treatments, ATP content was measured using a high-sensitivity commercial kit (Beyotime), which exhibited good linearity for ATP concentrations ranging from 0.1 nM to 10 μM. Briefly, supernatants were collected after the lysis of antler chondrocytes and then mixed with the detection solution for 3–5 min. Relative light units (RLUs) were measured using a luminometer (BioTek, Winooski, VT, USA). Finally, ATP content was calculated according to the standard curve.

### 2.20. Measurement of mPTP

After various treatments, antler chondrocytes were incubated with calcein AM and cobalt chloride for 30 min and then analyzed using flow cytometry or visualized after Hoechst staining under a fluorescence microscope, followed by calculation of fluorescence intensity using ImageJ software.

### 2.21. Intracellular and Mitochondrial ROS Detection

To determine the changes in intracellular ROS, antler chondrocytes were incubated with DCFH-DA (10 μM, Beyotime) for 20 min, and then analyzed using flow cytometry. For mtROS, after the addition of MitoSOX Red mitochondrial superoxide indicator (5 μM, Invitrogen) and incubation with Hoechst, antler chondrocytes were visualized under fluorescence microscopy, and fluorescence intensity was calculated using ImageJ software.

### 2.22. Measurement of SOD2 Activity

Antler chondrocytes were homogenized to obtain cell lysates. SOD2 activity was determined using a SOD Activity Assay Kit (Beyotime). Briefly, samples were incubated with SOD1 inhibitors A and B to inactivate SOD1, followed by the addition of WST-8/enzyme working solution and reaction initiator working solution. After incubation for 30 min, absorbance was measured at 450 nm using a microplate reader. SOD2 activity was calculated according to the operating instructions of the manufacturer.

### 2.23. Analysis of Mitochondrial Morphology

After the introduction of TGM2 overexpression and mito-DsRed plasmid (Addgene, #55838), antler chondrocytes were treated with PA for 12 h and then photographed under fluorescence microscope. Images were analyzed using ImageJ software to determine the changes in mitochondrial network morphology.

### 2.24. Analysis of Mitochondrial Redox Potential

After the introduction of TGM2 overexpression and mito-roGFP plasmid (Addgene, #82407) into antler chondrocytes, accompanied by PA treatment, images were captured at 405 nm and 488 nm excitation and 510 nm emission using a confocal microscope and then visualized after the addition of pseudocolor using ImageJ software. Finally, mitochondrial oxidized (red) and reduced (green) status was determined by analyzing changes in the fluorescence ratio at 405 and 488 nm excitation.

### 2.25. Measurement of Mitochondrial Membrane Potential

To determine changes in the mitochondrial membrane potential, antler chondrocytes were incubated with JC-1 fluorescent probe (Beyotime) and then analyzed using flow cytometry. Concurrently, the mitochondrial membrane potential was visualized after incubation with TMRM fluorescence dye (Invitrogen) and subsequent Hoechst staining, followed by calculation of fluorescence intensity.

### 2.26. Measurement of Lipid Peroxidation

After various treatments, antler chondrocytes were incubated with C11-BODIPY581/591 (Sigma) for 20 min. Images were captured using a fluorescence microscope, followed by analysis of the green/red fluorescence ratio. Meanwhile, lysed chondrocytes were used to measure the variation in malondialdehyde (MDA) content according to the instructions of the corresponding commercial kits (Beyotime).

### 2.27. Chromatin Immunoprecipitation (ChIP)

After RIN1 treatment, ChIP assay was performed as previously described [32]. Briefly, antler chondrocytes were cross-linked with 1% formaldehyde and then fragmented by ultrasonication. Immunoprecipitation was performed using anti-RBPJ antibody or control IgG. After incubation with Protein A/G agarose beads, DNA was purified and analyzed by real-time PCR with specific primers. The primers used were as follows: 5′-GAGATTTCCCTGGTGGCTCA-3′ and 5′-GGATGGAAAGTGAAAGCCGC-3′.

### 2.28. Plasmid Construction and Site-Directed Mutagenesis

The TMTC4 promoter sequence (−1210 to −910) containing RBPJ binding sites was amplified using specific primers (5′-CTGCTCGAGGTGATACTGGGGGAAAAGCAG and 5′-CTGAAGCTTAGGGGAATCTTCTGGACCCAA) and cloned into the pGL6 luciferase reporter vector (Beyotime) to construct the wild-type pGL6-TMTC4 plasmid. Using this plasmid as the template, the corresponding mutant plasmid was generated by PCR-based site-directed mutagenesis with the following primers: 5′-CCTGGGTTAACAAGATCCTCTGGAGAAGGG and 5′-GAGGATCTTGTTAACCCAGGGATCGAAGCC.

### 2.29. Dual Luciferase Analysis

After the introduction of wild-type or mutant pGL6-TMTC4 plasmid, antler chondrocytes were treated with the RBPJ inhibitor RIN1 and then used to measure the luciferase activity by the dual luciferase reporter gene assay kit (Beyotime). The pRL-SV40 plasmid (Beyotime) was used for data normalization.

### 2.30. Detection of Mitophagosomes

After the introduction of EGFP-LC3 (Addgene, #21073) and mito-DsRed plasmids (Addgene, #55838) for 24 h, antler chondrocytes were subjected to serum starvation and then treated with PA in the absence or presence of intracellular Ca^2+^ chelator BAPTA, calcineurin inhibitor FK506 or NFATC inhibitor VIVIT. Finally, images were captured using a confocal microscope, followed by colocalization analysis of autophagosomes and mitochondria.

### 2.31. Measurement of Mitophagy Flux

After the introduction of COX8-EGFP-mCherry plasmid (Addgene, #78520) and treatment as previously described, images were captured using a confocal microscope, followed by the quantification of red plots per cell using ImageJ software.

### 2.32. Colocalization Analysis of Mitochondria and Lysosomes

After various treatments, antler chondrocytes were incubated with Mito-Tracker Green and Lyso-Tracker Red (50 nM, Beyotime) for 30 min. Images were captured using fluorescence microscopy. The PCC value was calculated using ImageJ software.

### 2.33. Measurement of Lysosomal Acidification

After various treatments, antler chondrocytes were incubated with LysoSensor Green DND-189 (1 μM, Yeasen) for 1 h and analyzed using flow cytometry. Then, chondrocytes were visualized after Hoechst staining under fluorescence microscopy, followed by analysis of fluorescence intensity.

### 2.34. Statistical Analysis

All experiments were performed with at least three independent biological replicates. Statistical differences between two groups were assessed by the independent-samples T test using GraphPad Prism software (version 8.0.1), while multiple comparisons were executed using one-way ANOVA with Tukey’s multiple comparisons test. Data were presented as mean ± SEM. *p* < 0.05 was considered statistically significant.

## 3. Results

### 3.1. Effects of PA on the Hypertrophy, Proliferation and Apoptosis of Antler Chondrocytes

**Figure 1:**
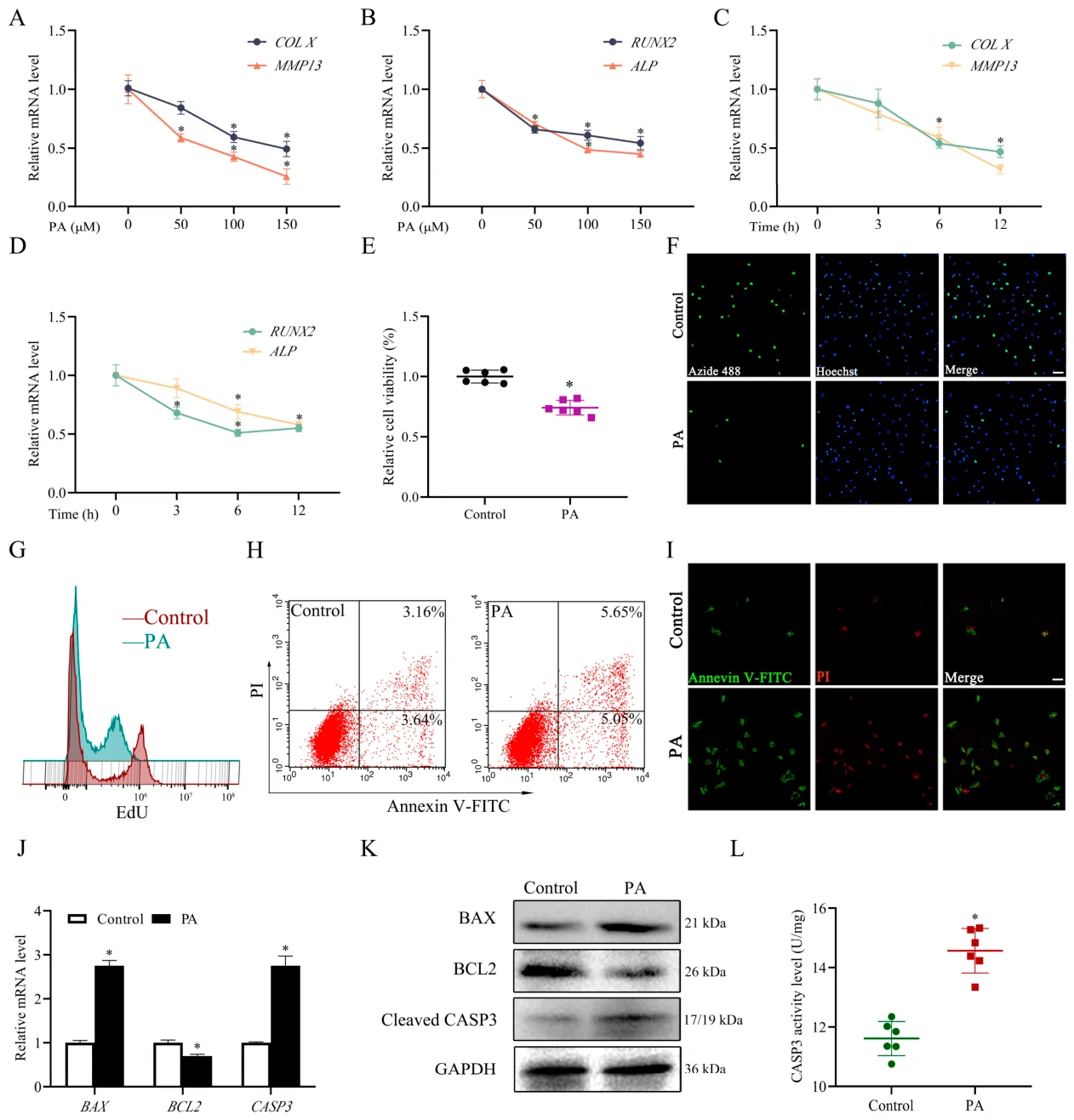
Effects of PA on hypertrophy, proliferation and apoptosis of antler chondrocytes. (**A**,**B**) Determination of *COL X*, *MMP13*, *RUNX2* and *ALP* expression after treatment with PA (50, 100 and 150 μM). *n* = 4. (**C**,**D**) Analysis of *COL X*, *MMP13*, *RUNX2* and *ALP* expression after treatment with PA (100 μM) for 3, 6 and 12 h. *n* = 4. (**E**) Detection of chondrocyte viability after treatment with PA for 12 h. *n* = 6. (**F**) Visualization of chondrocyte proliferation after treatment with PA. Scale bar, 50 μm. *n* = 6. (**G**) Flow cytometry analysis of chondrocyte proliferation after treatment with PA. *n* = 3. (**H**) Flow cytometry analysis of chondrocyte apoptosis after treatment with PA. *n* = 3. (**I**) Visualization of chondrocyte apoptosis after treatment with PA. *n* = 6. (**J**) Regulation of PA on the expression of *BCL2, BAX* and *CASP3* mRNA. *n* = 4. (**K**). Regulation of PA on the expression of BCL2, BAX and cleaved CASP3 protein. *n* = 3. (**L**) Regulation of PA on CASP3 activity. *n* = 6. * *p* < 0.05 versus control.

To investigate the role of PA in the hypertrophy of antler chondrocytes, we analyzed its effects on the expression of type X collagen (*COL X*), matrix metalloproteinase 13 (*MMP13*), runt-related transcription factor 2 (*RUNX2*) and alkaline phosphatase (*ALP*), which are well-established markers for hypertrophic chondrocytes [26]. Application of PA inhibited the expression of *COL X*, *MMP13*, *RUNX2* and *ALP*, followed by the most obvious effects at 100 and 150 μM (Figure 1A,B). Simultaneously, PA time-dependently decreased the mRNA levels of the above genes, reaching their minimum at 12 h (Figure 1C,D). Consistently, PA inhibited the proliferation of antler chondrocytes and reduced the number of EdU-positive chondrocytes (Figure 1E–G). Conversely, administration of PA resulted in obvious elevation of the chondrocyte apoptosis rate and a remarkable decline in anti-apoptotic B cell leukemia/lymphoma 2 (BCL2) mRNA and protein (Figure 1H–K). Moreover, PA up-regulated the expression of pro-apoptotic BCL2-associated X (BAX) and induced the cleavage of CASP3, with a concomitant increase in CASP3 activity and *CASP3* mRNA (Figure 1J–L).

### 3.2. PA Activated NOTCH1 Signaling to Control the Hypertrophy, Proliferation and Apoptosis of Antler Chondrocytes

**Figure 2:**
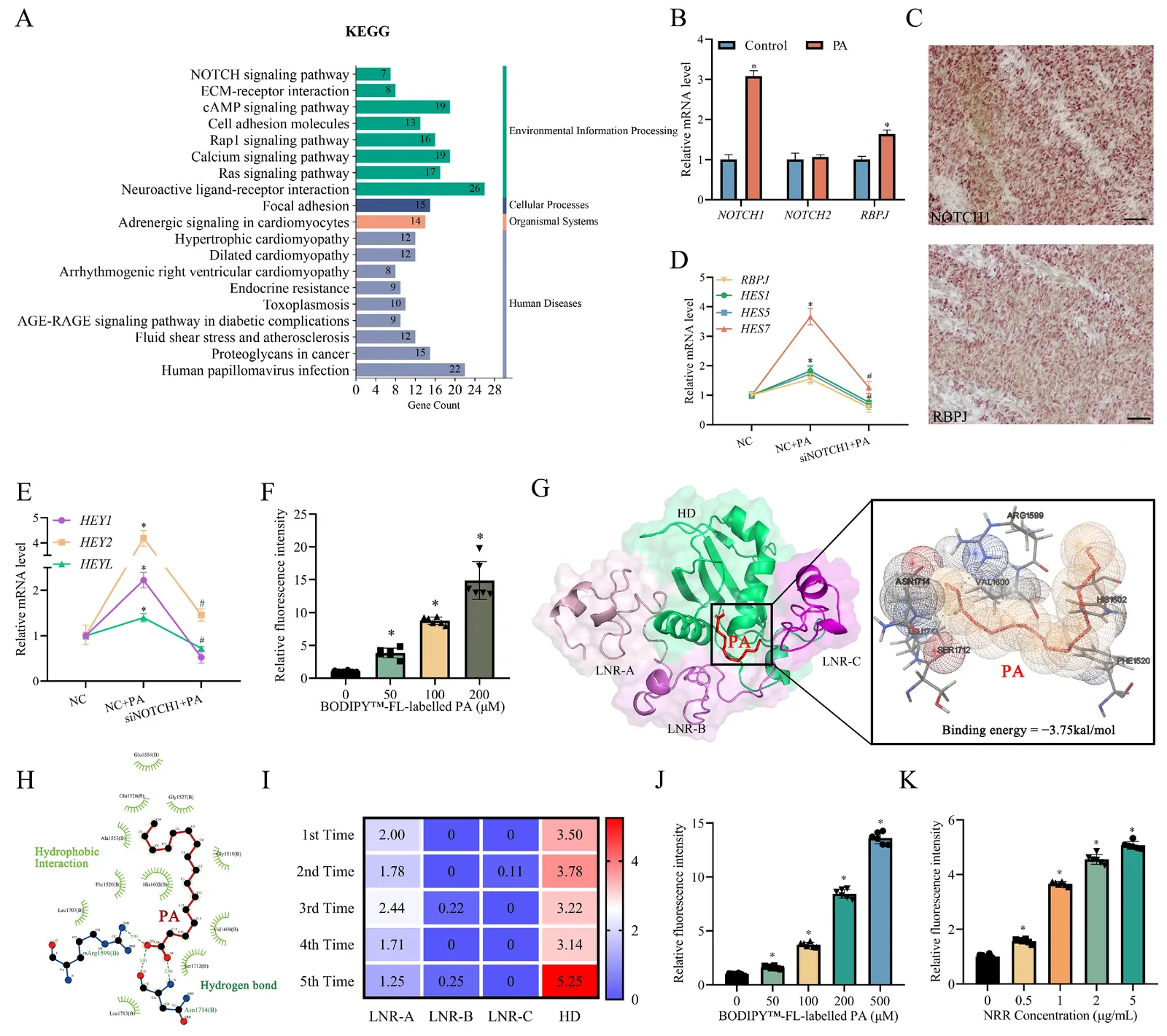
PA activated NOTCH1 signaling. (**A**) KEGG analysis of HEGs in antler cartilage layers. (**B**) Regulation of PA on the expression of *NOTCH1*, *NOTCH2* and *RBPJ* mRNA. *n* = 3. (**C**) In situ hybridization analysis of NOTCH1 and RBPJ expression in antler cartilage. Scale bar, 60 μm. *n* = 3. (**D**,**E**) Determination of *RBPJ*, *HES1*, *HES5*, *HES7*, *HEY1*, *HEY2* and *HEYL* mRNA expression after introduction of NOTCH1 siRNA and addition of PA. siNOTCH1, NOTCH1 siRNA. *n* = 3. (**F**) Analysis of binding between PA and NOTCH1. *n* = 6. (**G**) Molecular docking analysis of binding poses between PA and NRR of NOTCH1. (**H**) Two-dimensional diagram showing the binding of PA and NRR of NOTCH1. (**I**) Average number of PA molecules interacting with amino acid residues in LNR and HD of NOTCH1 at 5 independent repeats. (**J**) Analysis of binding between PA and NRR of NOTCH1 at a constant NRR concentration of 1 μg/mL. *n* = 6. (**K**) Analysis of binding between PA and NRR of NOTCH1 at a constant PA concentration of 100 μM. *n* = 6. * *p* < 0.05 versus control or NC, ^#^ *p* < 0.05 versus PA or NC plus PA treatment.

Bulk RNA-Seq analysis of antler cartilage layers revealed the enrichment of HEGs in the NOTCH and calcium signaling pathways and neuroactive ligand–receptor interactions, among others (Figure 2A). Given the absence of NOTCH3 and NOTCH4 in antler chondrocytes [33], the effects of PA on NOTCH1 and NOTCH2 expression were examined. The results reveal that PA increased the expression of *NOTCH1* and *RBPJ*, while it did not alter the expression of *NOTCH2* (Figure 2B). In situ hybridization results exhibit the obvious localization of *NOTCH1* and *RBPJ* in antler chondrocytes (Figure 2C). Meanwhile, application of PA elevated the expression of hes family bHLH transcription factor 1 (*HES1*), *HES5*, *HES7*, hes-related family bHLH transcription factor with YRPW motif 1 (*HEY1*), *HEY2* and *HEYL*, the known NOTCH signaling targets (Figure 2D,E and Figure S1A–C). However, silencing of NOTCH1 or addition of γ-secretase inhibitor DAPT counteracted the induction of PA on the above genes (Figure 2D,E and Figure S1A–D). Similarly, PA enhanced the expression of nuclear N1ICD and RBPJ protein, but this enhancement was neutralized after the introduction of NOTCH1 siRNA or treatment with DAPT (Figure S1E,F). Further analysis indicated the combination of PA and NOTCH1 (Figure 2F). A previous study showed that the extracellular domain of NOTCH1 contains EGF-like repeats and a NRR composed of three cysteine-rich Lin12-Notch repeats (LNRs) and a heterodimerization domain (HD) [34]. Because the NOTCH1 extracellular domain in *Homo sapiens* and *Cervus elaphus* exhibited high homology (Figure S1G–I), the 3D structures of human EGF-like repeats and NRR were used to perform molecular docking. The results suggest that PA might interact with the HD of the NRR via hydrogen and hydrophobic interaction, with a total binding energy of −3.75 kcal/mol, but not with the EGF-like repeats (Figure 2G–I and Figure S1J). Consistently, PA exhibited concentration-dependent affinity with NRR protein (Figure 2J,K). Moreover, transfection with NOTCH1 siRNA and replenishment of DAPT neutralized the repression of PA on the hypertrophy and proliferation of antler chondrocytes and protected the chondrocytes against apoptosis in the context of PA (Figures S2A–H and S3A–J).

### 3.3. PA Restricted the Uptake of ER Ca^2+^ and Enhanced Cytosolic Ca^2+^ Levels via RBPJ-Targeted TMTC4

**Figure 3:**
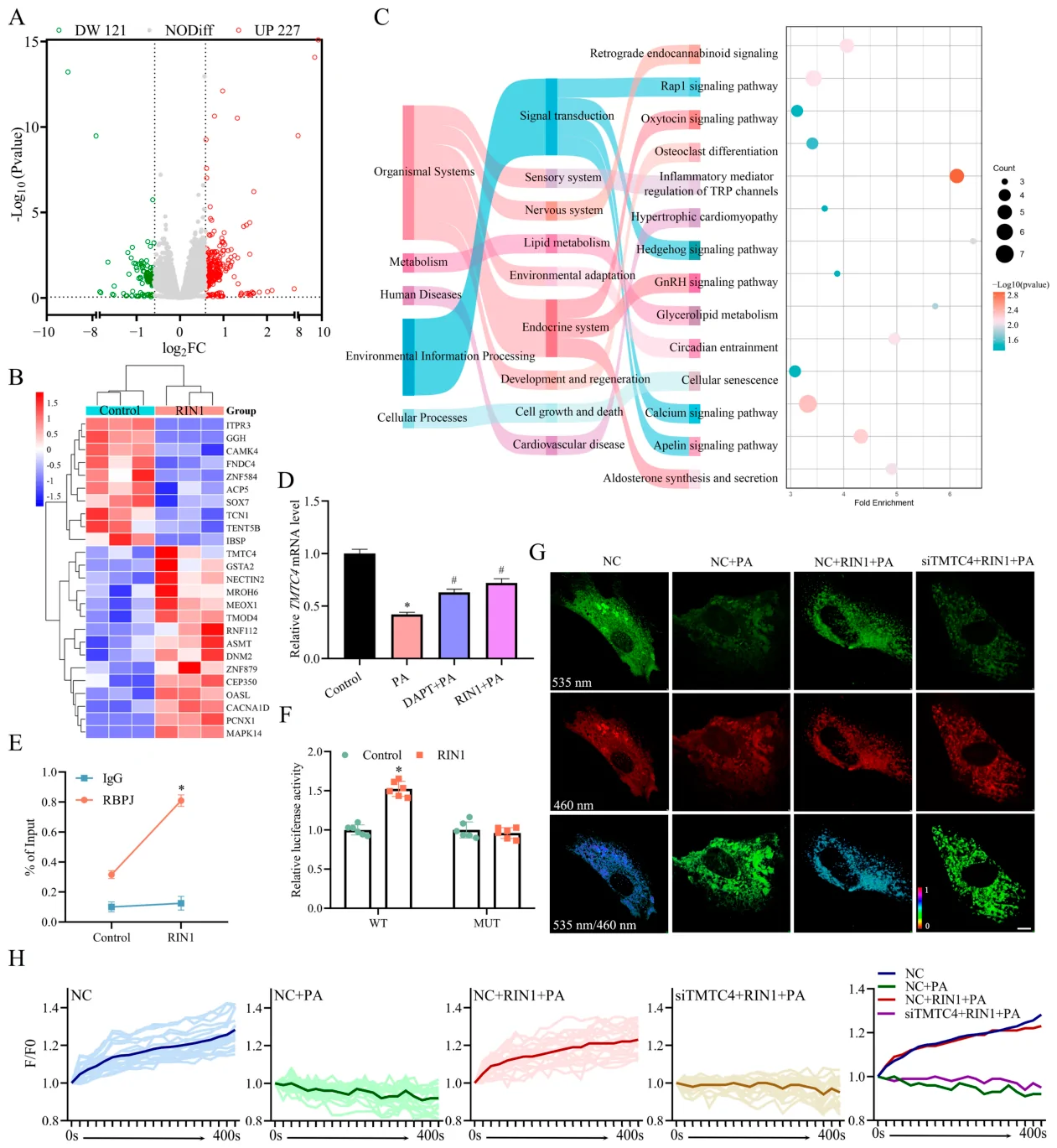
PA restricted the uptake of ER Ca^2+^ via RBPJ-targeted TMTC4. (**A**) Volcano plot displays DEGs after treatment with RBPJ inhibitor RIN1. (**B**) Heatmap exhibits the top 25 DEGs after treatment with RIN1. (**C**) KEGG analysis of DEGs after treatment with RIN1. (**D**) Regulation of PA on *TMTC4* expression in the absence or presence of DAPT and RIN1. *n* = 3. (**E**) ChIP analysis of binding between the RBPJ and TMTC4 promoter regions. *n* = 3. (**F**) Assessment of luciferase activity after introduction of wild-type or mutant pGL6-TMTC4 plasmid, followed by the addition of RIN1. *n* = 6. (**G**) Visualization of ER Ca^2+^ variation after introduction of pcDNA-D1ER plasmid and subsequent treatment with PA in the presence or absence of RIN1 and TMTC4 siRNA. siTMTC4, TMTC4 siRNA. Scale bar, 10 μm. *n* = 3. (**H**) Analysis of ER Ca^2+^ uptake after above treatment. * *p* < 0.05 versus control, ^#^ *p* < 0.05 versus PA treatment.

RNA-Seq analysis revealed that after exposure to RBPJ inhibitor RIN1, 227 DEGs were up-regulated, while 121 DEGs, which included TMTC4, were down-regulated (Figure 3A,B). According to KEGG analysis, DEGs were enriched in the calcium signaling pathway, osteoclast differentiation, and other related pathways. (Figure 3C). GO analysis indicated the enrichment of DEGs in calcium ion transport, ossification, and other related pathways (Figure S4A). Application of PA attenuated the expression of *TMTC4* in antler chondrocytes, but this attenuation was reversed by knockdown of NOTCH1 or the addition of γ-secretase inhibitor DAPT and RBPJ inhibitor RIN1 (Figure 3D and Figure S4B). Bioinformatic analysis of the TMTC4 promoter region revealed the presence of a potential RBPJ binding site (Figure S4C), implying that TMTC4 may be a direct target of RBPJ. As expected, RBPJ bound to the TMTC4 promoter region (Figure 3E). Moreover, RIN1 increased luciferase activity after introduction of the TMTC4 promoter reporter plasmid, whereas mutation of this binding site abolished the above effect (Figure 3F). Since TMTC4 is involved in maintaining homeostasis of intracellular Ca^2+^ through its interaction with sarcoplasmic/endoplasmic reticulum calcium ATPase 2 (SERCA2), a Ca^2+^ reuptake pump in ER [35], the expression of *SERCA2* was examined and the changes in Ca^2+^ in the ER and cytosol were analyzed. The results indicate that PA diminished the expression of *SERCA2*, preventing the uptake of Ca^2+^ into ER and resulting in the decline of ER Ca^2+^ levels and accumulation of cytosolic Ca^2+^ (Figure 3G,H and Figure S4D–F). Addition of RIN1 restored ER Ca^2+^ levels, improved *SERCA2* expression and neutralized the elevation of cytosolic Ca^2+^ in the context of PA, but these effects were counteracted after the introduction of TMTC4 siRNA (Figure 3G,H and Figure S4D–G).

### 3.4. PA Disrupted MAM Function Through Ca^2+^-Mediated PPP3CB-NFATC2 Pathway with TGM2 Involvement

**Figure 4:**
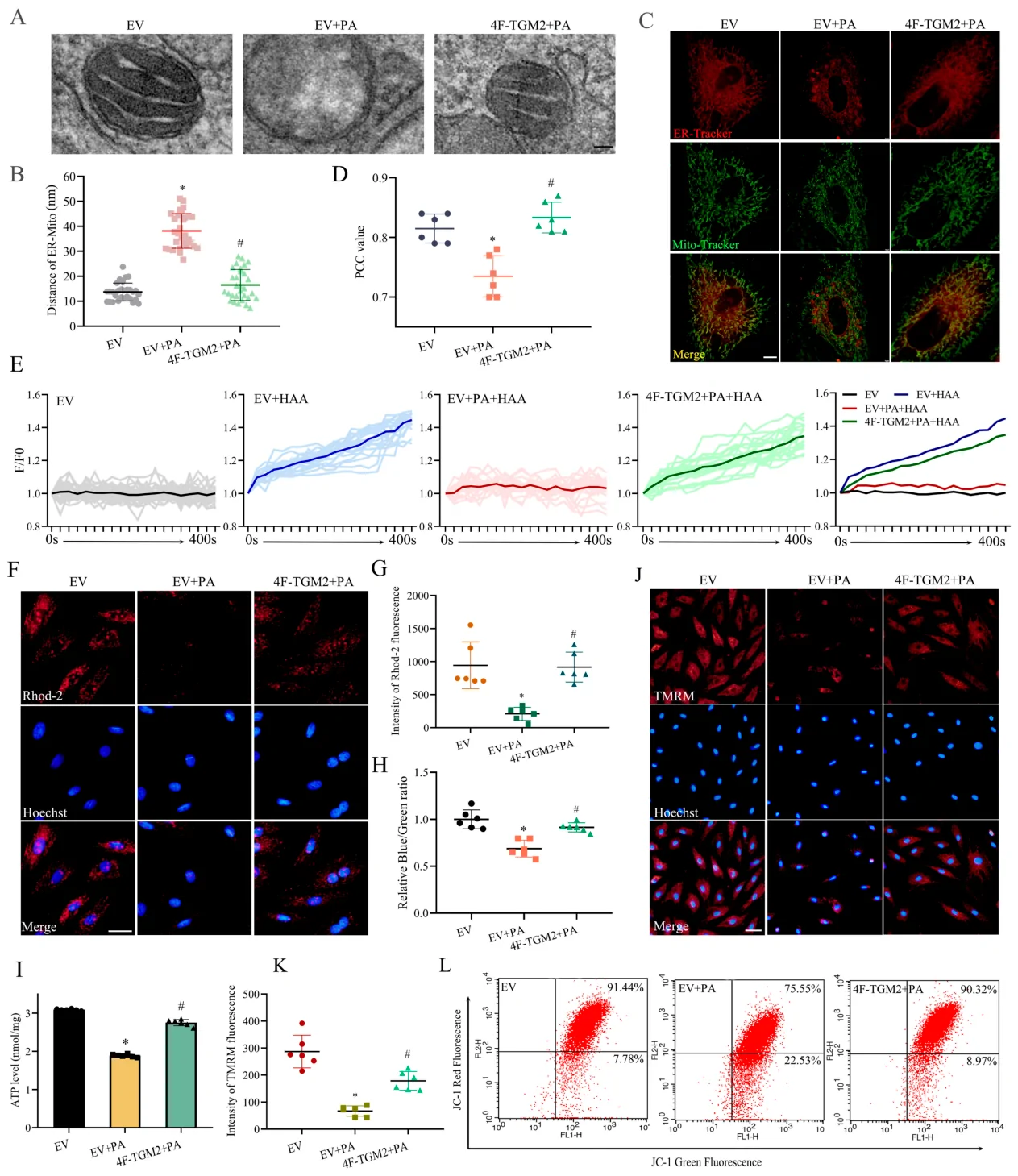
PA disrupted MAM function and then induced mitochondrial dysfunction via TGM2. (**A**,**B**) TEM analysis of ER-mitochondria distance after introduction of TGM2 overexpression plasmid and addition of PA. Scale bar, 50 nm. *n* = 3. (**C**,**D**) Visualization of ER-mitochondria contact sites and assessment of PCC values after above treatment followed by incubation with ER-Tracker Red and Mito-Tracker Green. EV, empty pCMV-4 × Flag vector; 4F-TGM2, TGM2 overexpression plasmid. Scale bar, 10 μm. *n* = 3. (**E**) Analysis of MAM function by measuring mitochondrial Ca^2+^ dynamics after introduction of TGM2 overexpression and mito-GEM-GECO1 plasmids and treatment with PA, followed by calculation of the blue/green fluorescent ratio under stimulation with histamine and ATP. HAA, histamine and ATP. (**F**,**G**) Visualization of mitochondrial Ca^2+^ variation after various treatments and incubation with Rhod-2 AM, and calculation of fluorescence intensity. Scale bar, 50 μm. *n* = 6. (**H**) Measurement of mitochondrial Ca^2+^ levels. *n* = 6. (**I**) Measurement of ATP content after various treatments. *n* = 6. (**J**,**K**) Visualization of mitochondrial membrane potential after various treatments and incubation with TMRM fluorescence dye, followed by calculation of fluorescence intensity. Scale bar, 50 μm. *n* = 6. (**L**) Flow cytometry analysis of mitochondrial membrane potential change behind various treatments and incubation with JC-1 fluorescent probe. *n* = 3. * *p* < 0.05 versus EV, ^#^ *p* < 0.05 versus EV plus PA treatment.

After exposure to PA, TEM analysis revealed an increased distance between ER and mitochondria in antler chondrocytes (Figure 4A,B). Consistent with this observation, the colocalization of ER and mitochondria was obviously reduced, together with a decline in the PCC value (Figure 4C,D), indicating disruption of MAMs. To further confirm the changes in MAMs, the SPLICS Mt-ER Long P2A plasmid was transfected into antler chondrocytes, followed by the addition of PA. The results reveal that PA attenuated the fluorescence intensity of the SPLICS sensor (Figure S4H,I). MAM function was assessed by monitoring mitochondrial Ca^2+^ dynamics upon stimulation with histamine and ATP, which promoted Ca^2+^ transfer from the ER to mitochondria via MAMs [30,31]. In antler chondrocytes, histamine and ATP increased the blue/green fluorescent ratio, indicating enhanced MAM-mediated transfer of ER Ca^2+^ into the mitochondria (Figure 4E). But application of PA neutralized the stimulatory effects of histamine and ATP on MAM function (Figure 4E). It has been previously reported that TGM2 is required to maintain the structure and function of MAMs [36]. The addition of PA diminished the expression of *TGM2*, while overexpression of TGM2 improved the disruption of MAMs elicited by PA (Figure 4A–E, Figures S4H,I and S5A–C). Additionally, overexpression of TGM2 attenuated the inhibitory effects of PA on chondrocyte hypertrophy and proliferation and protected chondrocytes against PA-induced apoptosis (Figure S5D–N).

The underlying regulatory mechanism of PA on TGM2 was then elucidated. As described above, PA induced the accumulation of cytosolic Ca^2+^, which might activate calcineurin, comprising a catalytic subunit encoded by the protein phosphatase 3 catalytic subunit alpha (PPP3CA), PPP3CB or PPP3CC gene and a regulatory subunit encoded by the PPP3R1 gene, thereby promoting transcriptional activity of NFATC [37]. PA augmented the expression of *PPP3CB* and *NFATC2*, with no significant changes in *PPP3CA*, *PPP3CC*, *PPP3R1*, *NFATC1*, *NFATC3* or *NFATC4* expression. However, this augmentation was weakened after attenuation of NOTCH1 or addition of DAPT, RIN1 and intracellular Ca^2+^ chelator BAPTA (Figure S6A–C). Silencing of TMTC4 counteracted the effects of RIN1 on *PPP3CB* and *NFATC2* in the context of PA (Figure S6D). Additionally, addition of calcineurin inhibitor FK506 or attenuation of *PPP3CB* suppressed the up-regulation of *NFATC2* by PA (Figure S6E,F). Further analysis demonstrated that knockdown of PPP3CB or NFATC2 reversed the effects of chondrocyte hypertrophy, proliferation and apoptosis induced by PA (Figures S6G–K and S7A–F). Moreover, addition of BAPTA, FK506 or NFATC inhibitor VIVIT abrogated the induction of PA on *TGM2* (Figure S5A), indicating the involvement of the Ca^2+^-mediated PPP3CB-NFATC2 pathway in the regulation of PA on TGM2.

### 3.5. PA Induced Mitochondrial Dysfunction by Reducing Mitochondrial Ca^2+^ Content Through MAM Disruption

In antler chondrocytes, PA diminished the fluorescence intensity of Rhod-2 and reduced the blue/green fluorescence ratio after introduction of mito-GEM-GECO1 plasmid, indicating a decline of mitochondrial Ca^2+^ content (Figure 4F–H). It is well established that mitochondrial Ca^2+^ is a crucial regulator of oxidative phosphorylation [38]. Application of PA restrained the chondrocyte production of ATP (Figure 4I). Mitochondrial membrane potential is a universal indicator of mitochondrial function [39]. PA depolarized the mitochondrial membrane potential, as evidenced by the attenuation of TMRM fluorescence intensity (Figure 4J,K). Flow cytometry analysis further substantiated the repression of PA on mitochondrial membrane potential, as evidenced by the reduced red/green fluorescence ratio after incubation with the JC-1 fluorescent probe (Figure 4L). Concurrently, PA disrupted the mitochondrial network, along with an obvious elevation in mitochondrial individual and network counts and decreases in mean branch length and mean network size (branches) (Figure S8A–E). However, overexpression of TGM2, which restored MAM function, ameliorated the insufficiency of mitochondrial Ca^2+^ elicited by PA and neutralized aberrant ATP production and mitochondrial depolarization while improving the mitochondrial network in the context of PA (Figure 4F–L and Figure S8A–E).

### 3.6. PA Inhibited Mitophagy via Ca^2+^-Mediated PPP3CB-NFATC2 Pathway

**Figure 5:**
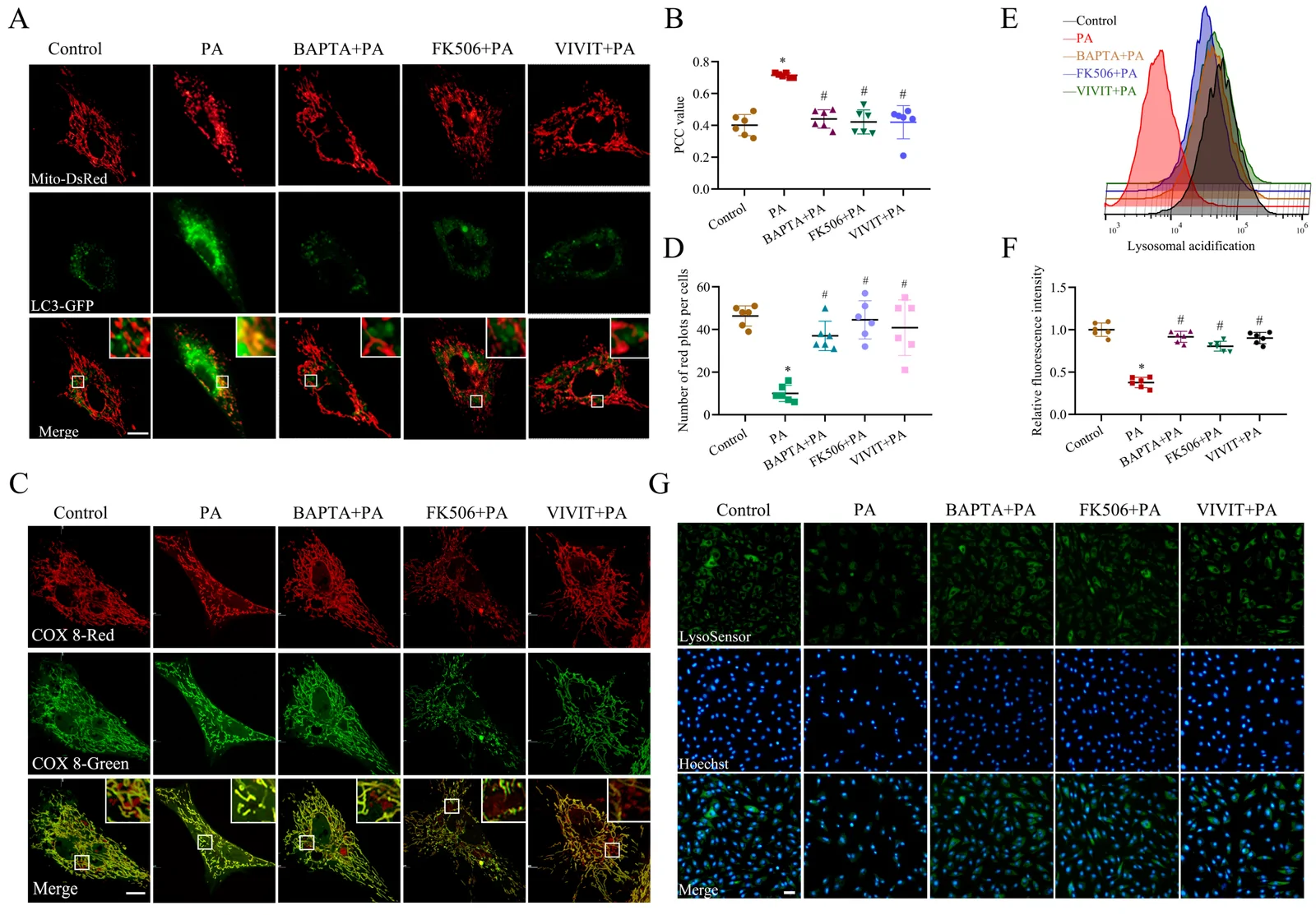
PA inhibited mitophagy via Ca^2+^-mediated PPP3CB-NFATC2 pathway. (**A**) Analysis of mitophagosomes after introduction of EGFP-LC3 and mito-DsRed plasmids in the absence or presence of intracellular Ca^2+^ chelator BAPTA, calcineurin inhibitor FK506 and NFATC inhibitor VIVIT. The white box marks the region magnified in the upper-right inset. Scale bar, 10 μm. (**B**) Assessment of PCC value using ImageJ software. *n* = 6. (**C**) Visualization of mitophagy flux after introduction of COX8-EGFP-mCherry plasmid, together with the aforementioned treatment. The white box marks the region magnified in the upper-right inset. Scale bar, 10 μm. (**D**) The number of red plots per cell using ImageJ software. *n* = 6. (**E**) Flow cytometry assay for lysosomal acidification following the above treatments. *n* = 3. (**F**,**G**) Visualization of lysosomal acidification and analysis of fluorescence intensity after aforementioned treatment and subsequent incubation with LysoSensor Green DND-189 fluorescent probe. Scale bar, 50 μm. *n* = 6. * *p* < 0.05 versus control, ^#^ *p* < 0.05 versus PA treatment.

Mitophagy was crucial to mitochondrial quality control and removed dysfunctional mitochondria from lysosomes [22]. PA induced the formation of mitophagosomes, as evidenced by the elevated colocalization between autophagosomes and mitochondria, while restricting mitophagy flux upon serum starvation (Figure 5A–D). Further analysis revealed that PA attenuated the colocalization of mitochondria and lysosomes, followed by an obvious reduction in the PCC value (Figure S8F,G). Consistently, PA weakened lysosomal acidification, as indicated by the weakened fluorescence signal after incubation with LysoSensor Green DND-189 (Figure 5E–G). As mentioned above, PA activated the Ca^2+^-PPP3CB-NFATC2 pathway. It was next explored whether this pathway was involved in the regulation of PA on mitophagy. These results demonstrate that the addition of intracellular Ca^2+^ chelator BAPTA, calcineurin inhibitor FK506 and NFATC inhibitor VIVIT counteracted the induction of PA on mitophagosomes and alleviated the defective mitophagy flux caused by PA (Figure 5A–D). Consistently, treatment with FK506 and VIVIT reversed the decreased colocalization of mitochondria with lysosomes and restored lysosomal acidification upon PA stimulation (Figure 5E–G and Figure S8F,G).

### 3.7. PA Facilitated Lipid Peroxidation by Inducing Leakage of mtROS into Cytosol Through mPTP Opening

**Figure 6:**
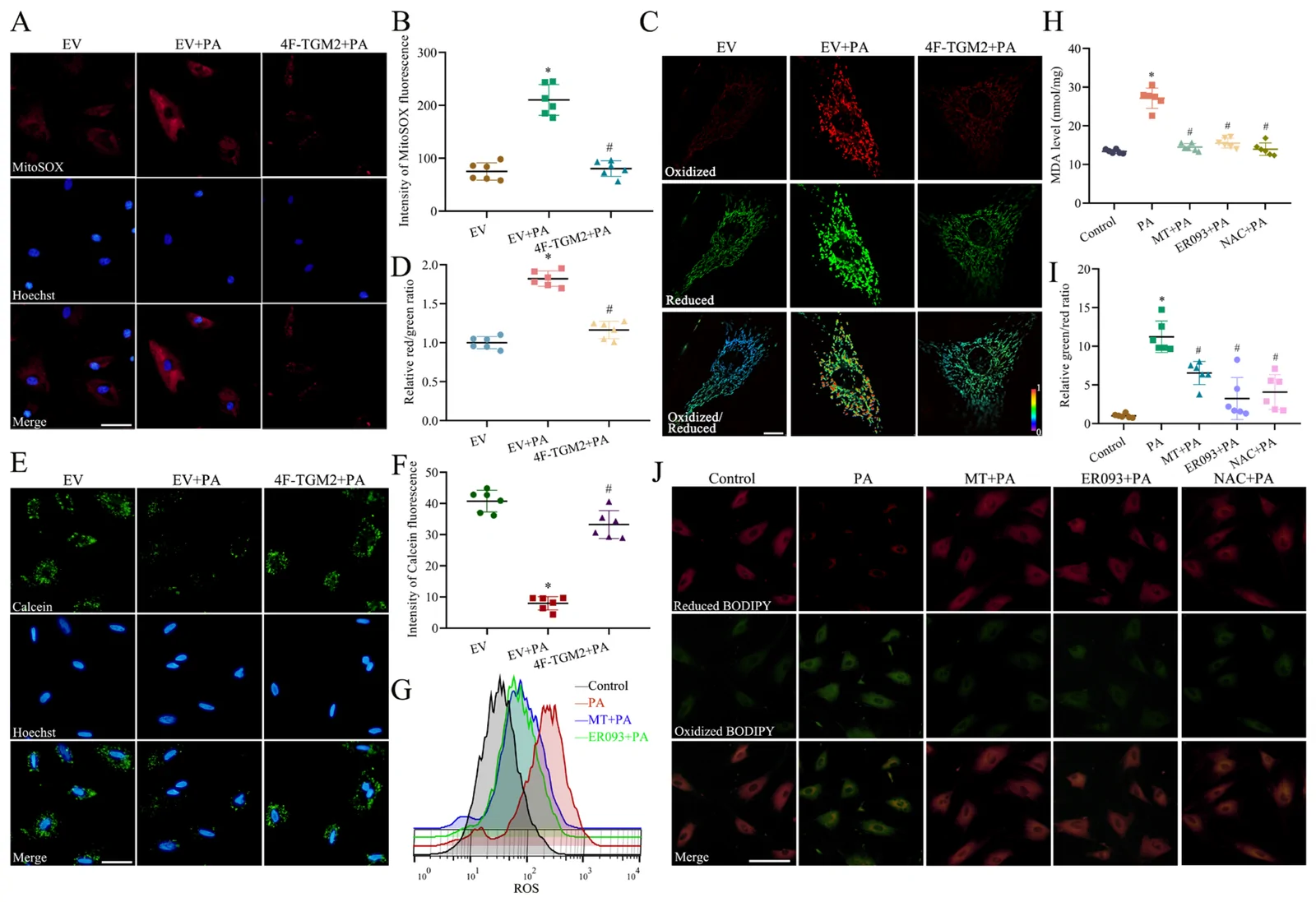
PA facilitated lipid peroxidation by inducing leakage of mtROS into cytosol through mPTP opening. (**A**,**B**) Visualization of mtROS variation and calculation of fluorescence intensity after introduction of TGM2 overexpression plasmid and addition of PA, followed by the incubation with MitoSOX Red mitochondrial superoxide indicator. Scale bar, 50 μm. *n* = 6. (**C,D**) Measurement of mitochondrial redox potential after introduction of mito-roGFP and TGM2 overexpression plasmids and addition of PA, followed by analysis of the fluorescence ratio at 405 and 488 nm excitation. Scale bar, 10 μm. *n* = 6. (**E**,**F**) Analysis of mPTP changes after introduction of TGM2 overexpression plasmid and addition of PA and subsequent incubation with calcein AM and cobalt chloride, followed by calculation of fluorescence intensity. Scale bar, 50 μm. *n* = 6. (**G**) Flow cytometry analysis of intracellular ROS after treatment with PA in the absence or presence of mitochondria-targeted ROS scavenger MT and subsequent incubation with DCFH-DA. *n* = 3. (**H**) Measurement of MDA content after treatment with PA in the presence or absence of MT, mPTP opening inhibitor ER093 and ROS scavenger NAC. *n* = 6. (**I,J**) Analysis of lipid peroxidation after above treatment and incubation with C11-BODIPY581/591, followed by analysis of the green/red fluorescence ratio. Scale bar, 50 μm. *n* = 6. * *p* < 0.05 versus control or EV, ^#^ *p* < 0.05 versus PA or EV plus PA treatment.

**Figure 7:**
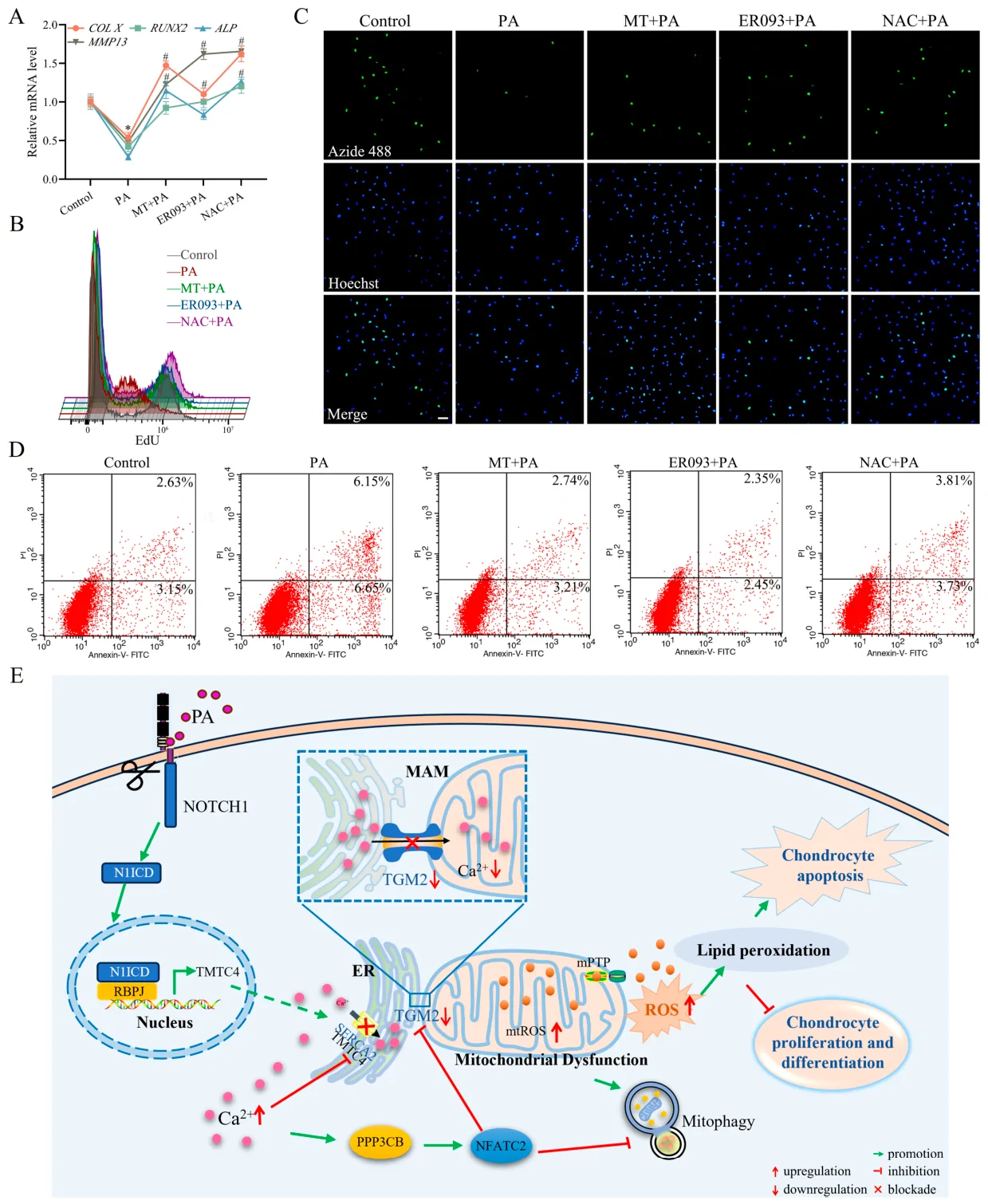
PA controlled the hypertrophy, proliferation and apoptosis of antler chondrocytes via mPTP-mediated mtROS leakage. (**A**) Determination of *COL X*, *MMP13*, *RUNX2* and *ALP* expression after treatment with PA in the absence or presence of MT, ER093 and NAC. *n* = 4. (**B**) Flow cytometry analysis of chondrocyte proliferation behind above treatment. *n* = 3. (**C**) Visualization of chondrocyte proliferation following above treatment. Scale bar, 50 μm. *n* = 3. (**D**) Flow cytometry analysis of chondrocyte apoptosis following above treatment. *n* = 3. (**E**) Schematic depiction of PA regulatory mechanism in antler chondrocytes. PA activated NOTCH1 signaling and prevented the transport of cytosolic Ca^2+^ to ER via RBPJ-targeted TMTC4, resulting in the accumulation of cytosolic Ca^2+^ and subsequent activation of the PPP3CB-NFATC2 pathway. This disrupted MAMs via TGM2, induced mitochondrial dysfunction accompanied by impaired mitophagy and facilitated leakage of mtROS through mPTP opening, ultimately leading to lipid peroxidation, defective hypertrophy and proliferation and increased apoptosis in antler chondrocytes. * *p* < 0.05 control, ^#^ *p* < 0.05 versus PA treatment.

Analysis of HEGs exhibited enrichment of response to redox state, positive regulation of chondrocyte differentiation, membrane depolarization, and more (Figure S9A). In antler chondrocytes, PA induced the accumulation of mtROS and put the mitochondria in an oxidized state, as indicated by an increased fluorescence ratio at 405 and 488 nm excitation after introduction of mito-roGFP plasmid (Figure 6A–D). Consistently, PA attenuated the activity of SOD2, an important antioxidant enzyme within the mitochondria (Figure S9B). But overexpression of TGM2 alleviated the mitochondrial oxidized potential together with an obvious reduction for mtROS and enhanced the activity of SOD2 in the context of PA. (Figure 6A–D and Figure S9B). Previous reports have demonstrated that mitochondria are the main sources of ROS, and their dysfunction can promote mtROS leakage into the cytosol via mPTP, resulting in lipid peroxidation [40,41]. Treatment with PA facilitated the opening of mPTP, but this effect was abrogated after transfection with TGM2 overexpression plasmid (Figure 6E,F). Meanwhile, application of PA enhanced the contents of lipid peroxidation marker MDA and intracellular ROS and gave rise to an increase in the green/red fluorescence ratio after incubation with C11-BODIPY581/591. In contrast, the addition of mitochondria-targeted ROS scavenger MT dose-dependently reduced mtROS levels and attenuated the accumulation of intracellular ROS upon PA stimulation (Figure 6G–J and Figure S9C). Similarly, mPTP opening inhibitor ER093 attenuated the induction of PA on intracellular ROS (Figure 6G). Moreover, the addition of MT or ER093 neutralized the effect of PA on lipid peroxidation, thereby improving hypertrophy and proliferation of antler chondrocytes and alleviating cellular apoptosis (Figure 6H–J, Figure 7A–D and Figure S9D–H). Furthermore, the addition of ROS scavenger NAC, which ameliorated the lipid peroxidation induced by PA, counteracted the inhibitory effect of PA on the hypertrophy and proliferation of antler chondrocytes and protected the PA-treated chondrocytes from apoptosis (Figure 6H–J, Figure 7A–D and Figure S9D–H).

## 4. Discussion

As the only mammalian appendages with full regenerative capacity, antlers undergo rapid regrowth driven by chondrocyte proliferation and differentiation, which critically modulates endochondral ossification. [2,3,42]. PA treatment restrained the proliferation of antler chondrocytes. A similar result was also observed in the human T/C-28a2 chondrocyte cell line [12]. Moreover, PA blocked their differentiation into hypertrophic chondrocytes. Despite their unprecedented growing rate, antler cartilage did not become cancerous but instead exhibited a high proportion of apoptotic chondrocytes, resulting in the removal of aberrant cells [2]. Addition of PA increased the apoptosis of antler chondrocytes. Consistently, administration of PA to male rats resulted in the degeneration of articular cartilage, along with a significant elevation in chondrocyte apoptosis [43]. Together, these observations indicate that PA plays an essential role in antler cartilage regeneration by regulating the hypertrophy, proliferation and apoptosis of chondrocytes.

NOTCH1 exerts an important function in regulating chondrogenesis, cartilage development and endochondral ossification. Upon ligand binding to its EGF-like repeats, its extracellular domain is cleaved at site 2 (S2) via ADAM metalloprotease, followed by subsequent S3 cleavage via γ-secretase, finally resulting in the release of N1ICD [34,44]. In antler chondrocytes, PA facilitated the elevation of N1ICD, whose activation resulted in skeletal malformation, along with the decline of hypertrophic chondrocyte number in the limb from day 18.5 embryo [44]. Silencing of NOTCH1 neutralized the inhibitory effect of PA on the hypertrophy and proliferation of antler chondrocytes and protected the PA-treated chondrocytes against apoptosis. Previous studies have demonstrated that in the absence of EGF-like repeats, mutation of HD and LNR-C activated NOTCH1 signaling [45,46]. Although molecular docking suggested that PA preferentially interacted with the HD region of the NRR, direct evidence for their binding requires further investigation. Following the release and nuclear translocation of N1ICD, it binds to RBPJ and then modulates the transcription of downstream target genes [34]. The present study has identified TMTC4 as a direct downstream target of RBPJ. In neonatal organotypic cultures, deletion of TMTC4 brought about the insufficiency of ER Ca^2+^ through its interaction with the ER Ca^2+^ reuptake pump SERCA2, resulting in an increase in intracellular Ca^2+^ [35]. Application of PA accelerated the decline of ER Ca^2+^, attenuated *SERCA2* expression and induced the accumulation of cytosolic Ca^2+^. In contrast, addition of the RBPJ inhibitor RIN1 counteracted the above effects, but this antagonistic role was neutralized after TMTC4 silencing. Taken together, these observations indicate that PA activates NOTCH1 signaling and reduces ER Ca^2+^ uptake via RBPJ-targeted TMTC4, ultimately causing an increase in cytosolic Ca^2+^.

Mitochondria have the ability to sequester cytosolic Ca^2+^ [47]. Intriguingly, PA diminished the content of mitochondrial Ca^2+^ in antler chondrocytes. It is well established that MAM is crucial to mitochondrial Ca^2+^ homeostasis, and its disruption contributes to a deficiency in mitochondrial Ca^2+^ levels [17,48]. Application of PA destroyed the structure of MAM and weakened its function in transferring Ca^2+^ into mitochondria. A previous study reported that deficiency of TGM2 disrupted MAMs and increased the distance between ER and mitochondria [36]. In antler chondrocytes, PA reduced the expression of *TGM2*, whereas overexpression of TGM2 alleviated disrupted MAMs induced by PA and concomitantly restored mitochondrial Ca^2+^ levels. Taken together, these data indicate that PA attenuates mitochondrial Ca^2+^ content by disrupting MAMs through TGM2.

Mitochondria are important organelles for intracellular energy production, and their dysfunction brings about cartilage matrix degradation, chondrogenesis disorder, chondrocyte apoptosis and senescence [49,50]. In hepatocytes and proximal tubular cells, PA impaired mitochondrial function via lipotoxicity [51,52]. The present study revealed that treatment with PA induced mitochondrial dysfunction through MAM-mediated mitochondrial Ca^2+^ insufficiency. Mitophagy might selectively remove dysfunctional mitochondria [22]. As expected, PA inhibited mitophagy flux and reduced the colocalization of mitochondria with lysosomes in antler chondrocytes. Lysosomal acidification is crucial to mitophagy [22]. Application of PA attenuated lysosomal acidification. These data reveal the inhibitory effect of PA on mitophagy. Consistently, impaired mitophagy was also observed in chondrocytes from osteoarthritic articular cartilage and resulted in cartilage degeneration, whereas improvement of mitophagy alleviated the symptoms of osteoarthritis in mice [23].

Further analysis revealed that dysfunctional mitochondria contribute to excessive production of mtROS [53]. PA promoted the production of mtROS in chondrocytes, attenuated SOD2 activity and kept the mitochondria in an oxidized state. It was previously reported that prolonged mPTP opening is involved in the leakage of mtROS into the cytosol [41]. Treatment with PA caused the opening of mPTP, whose blockage neutralized the induction of PA on intracellular ROS. It is well known that elevated ROS is deleterious to the extracellular matrix, mineralization proliferation and maturation of chondrocytes and leads to lipid peroxidation [40,54,55]. Given the association between lipid peroxidation and ferroptosis and the reported role of PA in inducing ferroptosis in osteoarthritis [56], the regulation of ferroptosis by PA warrants further investigation. The addition of ROS scavenger NAC mitigated lipid peroxidation and reversed the effects of hypertrophy, proliferation and apoptosis in antler chondrocytes in the context of PA. Together, these observations indicate that PA induces the mitochondrial dysfunction, subsequently promoting leakage of mtROS into the cytosol through mPTP opening and thereby facilitating lipid peroxidation. Although PA has been reported to induce ER stress in C2C12 myoblasts and hepatocytes [57,58], its effect on ER stress in antler chondrocytes remains to be fully elucidated. Therefore, the possibility that ER stress might act as a parallel or upstream event contributing to chondrocyte impairment cannot be excluded. Meanwhile, protein-level validation for several key proteins was limited in this study due to the lack of antibodies with sufficient cross-reactivity with deer antigens.

Collectively, PA restrained the hypertrophy and proliferation of antler chondrocytes and promoted the chondrocyte apoptosis. PA could activate NOTCH1 signaling and prevent the uptake of ER Ca^2+^ via RBPJ-targeted TMTC4, resulting in the accumulation of cytosolic Ca^2+^. This disrupted MAMs, induced mitochondrial dysfunction, inhibited mitophagy and facilitated the leakage of mtROS through mPTP, ultimately leading to lipid peroxidation, defective hypertrophy and proliferation and increased apoptosis in antler chondrocytes. However, as our conclusions are primarily based on in vitro experiments using isolated chondrocytes, further in vivo studies are required to validate the physiological relevance of these findings. The mechanism discovered in deer antlers may provide broader insights for understanding the regulation of cartilage regeneration and repair in other model animals, although cross-species extrapolation requires further investigation.

## Data Availability

The RNA-seq data presented in this study have been deposited in the NCBI Sequence Read Archive (SRA) database under accession number PRJNA1512457.

## Supplementary Materials

The following supporting information can be downloaded at https://www.mdpi.com/article/10.3390/antiox15091108/s1: Figure S1: Regulation of PA on NOTCH1 signaling and analysis of NOTCH1 extracellular domain sequences; Figure S2: PA controlled the hypertrophy and proliferation of antler chondrocytes via NOTCH1 signaling; Figure S3: PA controlled the hypertrophy and proliferation of antler chondrocytes via NOTCH1 signaling; Figure S14: Regulation of PA on MAM, cytosolic Ca^2+^ and TMTC4, SERCA2 and TGM2 expression; Figure S5: PA controlled hypertrophy, proliferation and apoptosis of antler chondrocytes via TGM2-mediated MAM; Figure S6: PA controlled the hypertrophy and proliferation of antler chondrocytes via Ca^2+^-mediated PPP3CB-NFATC2 pathway; Figure S7: PA controlled the apoptosis of antler chondrocytes via PPP3CB-NFATC2 pathway; Figure S8: Effects of PA on mitochondrial morphology and mitophagy; Figure S9: Effects of PA on the proliferation and apoptosis of antler chondrocytes via mPTP-mediated mtROS leakage; Table S1: Homologous siRNA sequences used in this study; Table S2: Primer sequences using for qPCR; Table S3: Primary antibodies for Western blotting.

## Abbreviations

PA, palmitic acid; ER, endoplasmic reticulum; MAM, mitochondria-associated ER membrane; N1ICD, NOTCH1 intracellular domain; RBPJ, recombination signal binding protein for immunoglobulin kappa J region; TMTC4, transmembrane O-mannosyltransferase targeting cadherins 4; TGM2, transglutaminase 2; mtROS, mitochondrial reactive oxygen species; mPTP, mitochondrial permeability transition pore; NAC, N-acetylcysteine; CASP3, caspase 3; MDA, malondialdehyde; COL X, type X collagen; MMP13, matrix metalloproteinase 13; RUNX2, runt-related transcription factor 2; ALP, alkaline phosphatase; BCL2, B cell leukemia/lymphoma 2; BAX, BCL2-associated X protein; HES1, hes family bHLH transcription factor 1; HEY1, hes-related family bHLH transcription factor with YRPW motif 1; SERCA2, sarcoplasmic/endoplasmic reticulum calcium ATPase 2; PPP3CA, protein phosphatase 3 catalytic subunit alpha.
