# Supplementary material for: Palmitic Acid Inhibits the Proliferation and Hypertrophy of Antler Chondrocytes by Disrupting Mitochondria-Associated Endoplasmic Reticulum Membrane Function
Source: Antioxidants (Basel). 2026 Sep 2;15(9):1108. doi: 10.3390/antiox15091108 (PMC13603583; doi:10.3390/antiox15091108)
Supplement: Supplementary file 1 [file antioxidants-15-01108-s001.zip › antioxidants-4425814-supplementary.pdf]

Palmitic Acid Inhibits the Proliferation and Hypertrophy of Antler Chondrocytes by Disrupting Mitochondria-Associated Endoplasmic Reticulum Membrane Function

Qiaoling Zhang†, Zhanqing Yang†, Xueyuan Yao, Yinfei Xing, Chenhao Wang, Baiyu Li, Zhanpeng Yue*, Bin Guo*

[1]College of Veterinary Medicine, Jilin University, Changchun, China

*Correspondence:

Zhan-Peng Yue and Bin Guo

Email: yuezp@jlu.edu.cn; guobin79@jlu.edu.cn

College of Veterinary Medicine, Jilin University, Changchun 130062, China

† These authors contributed equally to this work

Figure S1

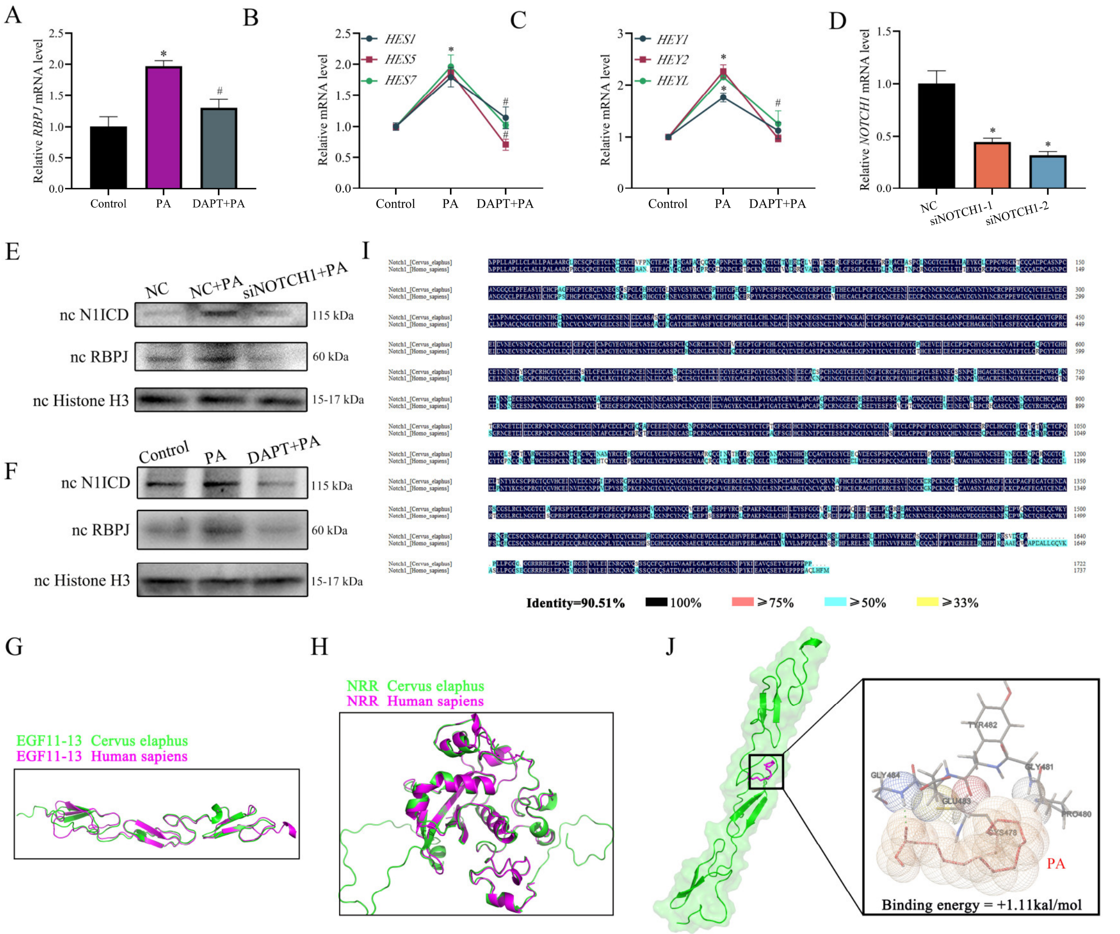

Figure S1. Regulation of PA on NOTCH1 signaling and analysis of NOTCH1 extracellular domain sequences. (A-C) Regulation of PA on the expression of *RBPJ*, *HES1*, *HES5*, *HES7*, *HEY1*, *HEY2* and *HEYL* mRNA in the absence or presence of γ-secretase inhibitor DAPT. *n*=3. **(D)** Determination of *NOTCH1* mRNA expression after introduction of NOTCH1 siRNAs. siNOTCH1, NOTCH1 siRNA. *n*=3. **(E and F)** Regulation of PA on the expression of nuclear N1ICD and RBPJ protein in the existence or not of NOTCH1 siRNA and DAPT. *n*=3. **(G and H)** Comparison of the 3D structures of EGF-like 11-13 and NRR of NOTCH1 extracellular domain between Homo sapiens and Cervus elaphus using PyMOL software. **(I)** Comparison of NOTCH1 extracellular domain sequences between Homo sapiens and Cervus elaphus using DNAMAN software. **(J)** Molecular docking analysis of PA and EGF-like repeats of NOTCH1. * P < 0.05 versus control or NC, # P < 0.05 versus PA treatment.

Figure S2

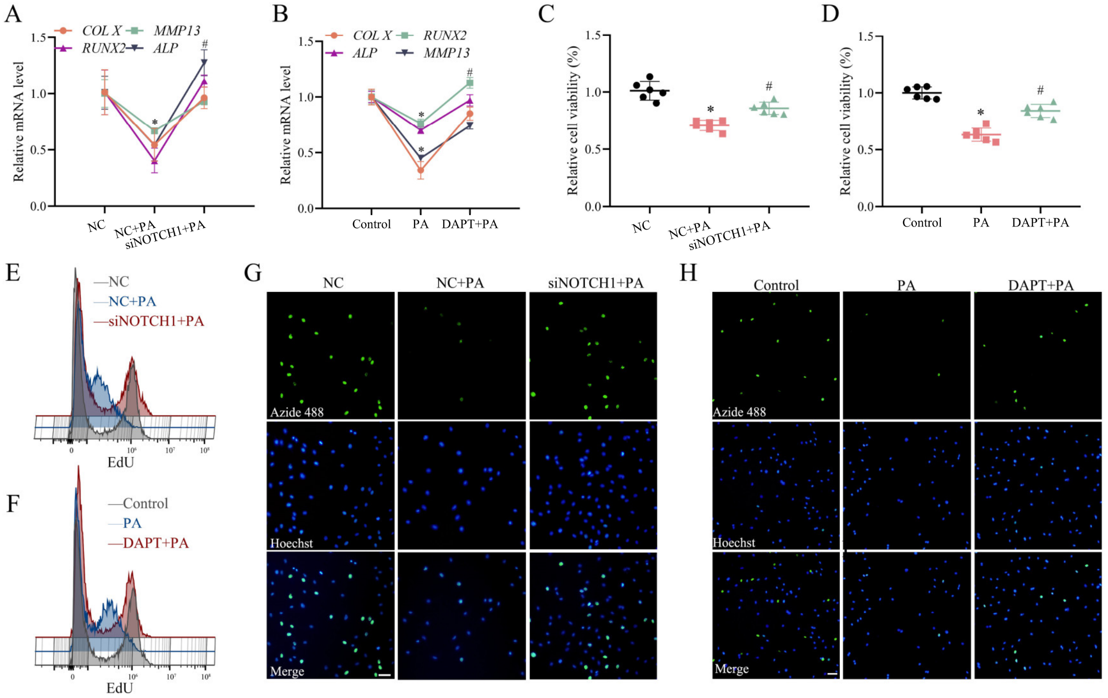

Figure S2. PA controlled the hypertrophy and proliferation of antler chondrocytes via NOTCH1 signaling. (A) Determination of *COL X*, *MMP13*, *RUNX2* and *ALP* expression after introduction of NOTCH1 siRNA and addition of PA. *n*=3. **(B)** Determination of *COL X*, *MMP13*, *RUNX2* and *ALP* expression after treatment with PA or DAPT plus PA. *n*=3. **(C and D)** Detection of chondrocyte viability after the above treatment. *n*=6. **(E and F)** Flow cytometry analysis of chondrocyte proliferation behind above treatment. *n*=3. **(G and H)** Visualization of chondrocyte proliferation behind above treatment. Scale bar, 50 µm. *n*=3. * $P < 0.05$ versus NC or control, # $P <$ 0.05 versus PA or NC plus PA treatment.

Figure S3

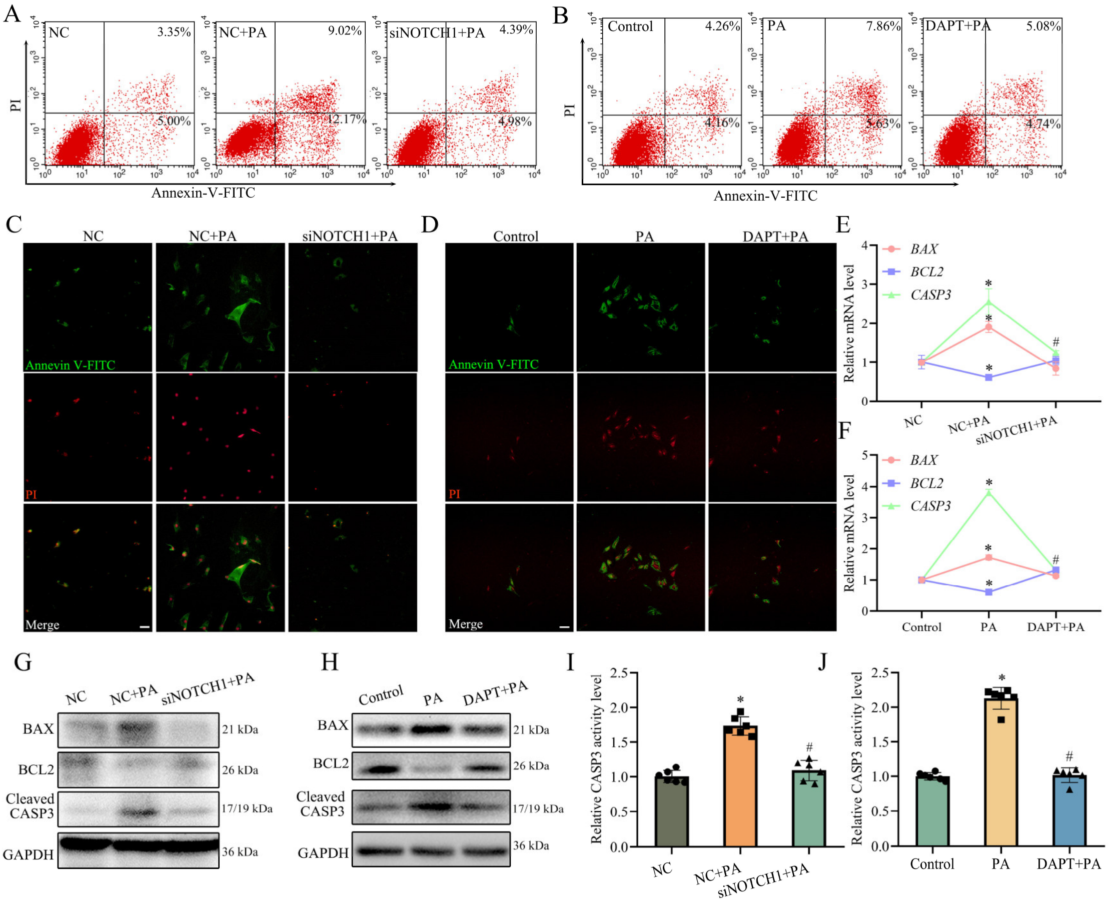

Figure S3. PA controlled the apoptosis of antler chondrocytes via NOTCH1 signaling. (A) Flow cytometry analysis of chondrocyte apoptosis after introduction of NOTCH1 siRNA and addition of PA. *n*=3. **(B)** Flow cytometry analysis of chondrocyte apoptosis after treatment with PA or DAPT plus PA. *n*=3. **(C and D)** Visualization of chondrocyte apoptosis behind above treatment. Scale bar, 50 μm. *n*=3. **(E and F)** Determination of *BCL2*, *BAX* and *CASP3* mRNA expression behind above treatment. *n*=4. **(G and H)** Detection of BCL2, BAX and cleaved CASP3 protein expression after the above treatment. *n*=3. **(I and J)** Measurement of CASP3 activity after the above treatment. *n*=6. * $P < 0.05$ versus NC or control, # $P < 0.05$ versus PA or NC plus PA treatment.

Figure S4

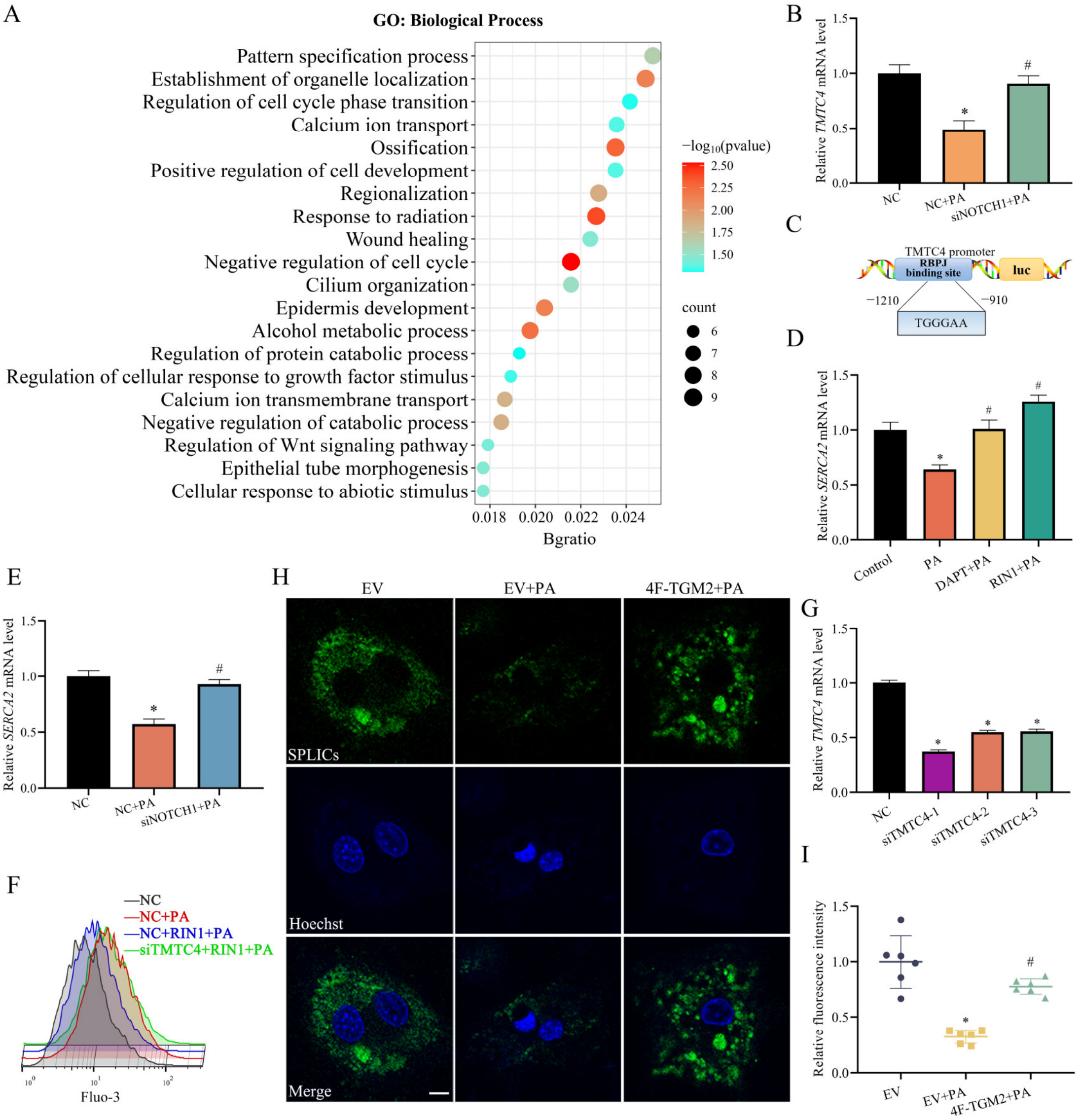

Figure S4. Regulation of PA on MAM, cytosolic Ca^{2+} and TMTC4, SERCA2 and TGM2 expression. (A) GO analysis of DEGs after treatment with RBPJ inhibitor RIN1. **(B)** Determination of *TMTC4* mRNA expression after introduction of NOTCH1 siRNA and addition of PA. n=3. **(C)** Schematic diagram showed the binding site of RBPJ in the TMTC4 promoter region. **(D)** Regulation of PA on *SERCA2* expression in the existence or not of DAPT and RIN1. *n*=3. **(E)** Determination of *SERCA2* mRNA expression after introduction of NOTCH1 siRNA. *n*=3. **(F)** Flow cytometry analysis of cytosolic Ca^{2+} variation after various treatment together with the subsequent incubation with Fluo-3 AM. *n*=3. **(G)** Determination of *TMTC4* mRNA expression after introduction of TMTC4 siRNAs. **si**TMTC4, TMTC4 siRNA. *n*=4. **(H)** Visualization of MAM variation after SPLICS Mt-ER Long P2A and TGM2 overexpression plasmids were co-

transfected into antler chondrocytes together with an addition of PA. EV, empty pCMV-4×Flag vector; 4F-TGM2, TGM2 overexpression plasmid. Scale bar, 10 μm. **(I)** Calculation of fluorescence intensity after above treatment. n=6. * $P < 0.05$ versus NC, EV or control. $^{\#} P < 0.05$ versus PA, NC or EV plus PA treatment.

Figure S5

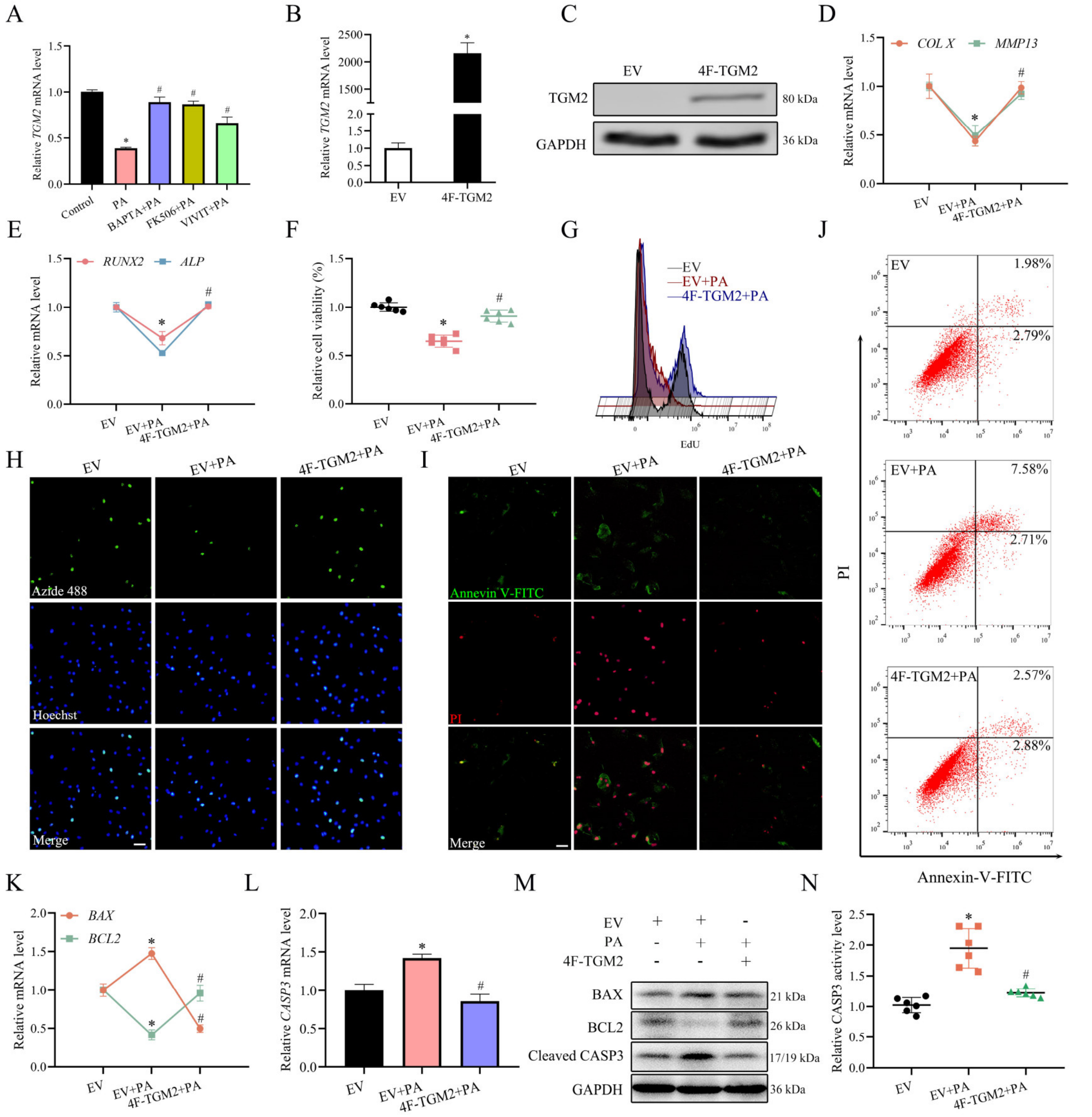

Figure S5. PA controlled the hypertrophy, proliferation and apoptosis of antler chondrocytes via TGM2-mediated MAM. (A) Determination of *TGM2* mRNA expression after treatment with PA in the absence or presence of intracellular Ca^{2+} chelator BAPTA, calcineurin inhibitor FK506 and NFATC inhibitor VIVIT. *n*=4. **(B and C)** Assessment of *TGM2* mRNA and protein expression after introduction of TGM2 overexpression plasmid. *n*=3. **(D)** Determination of *COL X* and *MMP13* expression after introduction of TGM2 overexpression plasmid and subsequent addition of PA. *n*=3. **(E)** Determination of *RUNX2* and *ALP* expression after the above treatment. *n*=3. **(F)** Detection of chondrocyte viability after the above treatment. *n*=6. **(G)** Flow cytometry analysis of chondrocyte proliferation after the above treatment. *n*=3. **(H)** Visualization of chondrocyte proliferation after the above treatment. Scale bar, 50 μm. *n*=3. **(I)** Visualization of chondrocyte

apoptosis after the above treatment. Scale bar, 50 μm. *n*=3. **(J)** Flow cytometry analysis of chondrocyte apoptosis after the above treatment. *n*=3. **(K and L)** Determination of *BCL2*, *BAX* and *CASP3* mRNA expression after the above treatment. *n*=3. **(M)** Detection of BCL2, BAX and cleaved CASP3 protein expression after the above treatment. *n*=3. **(N)** Measurement of CASP3 activity behind above treatment. *n*=6. * $P < 0.05$ versus EV, # $P < 0.05$ versus EV plus PA treatment.

Figure S6

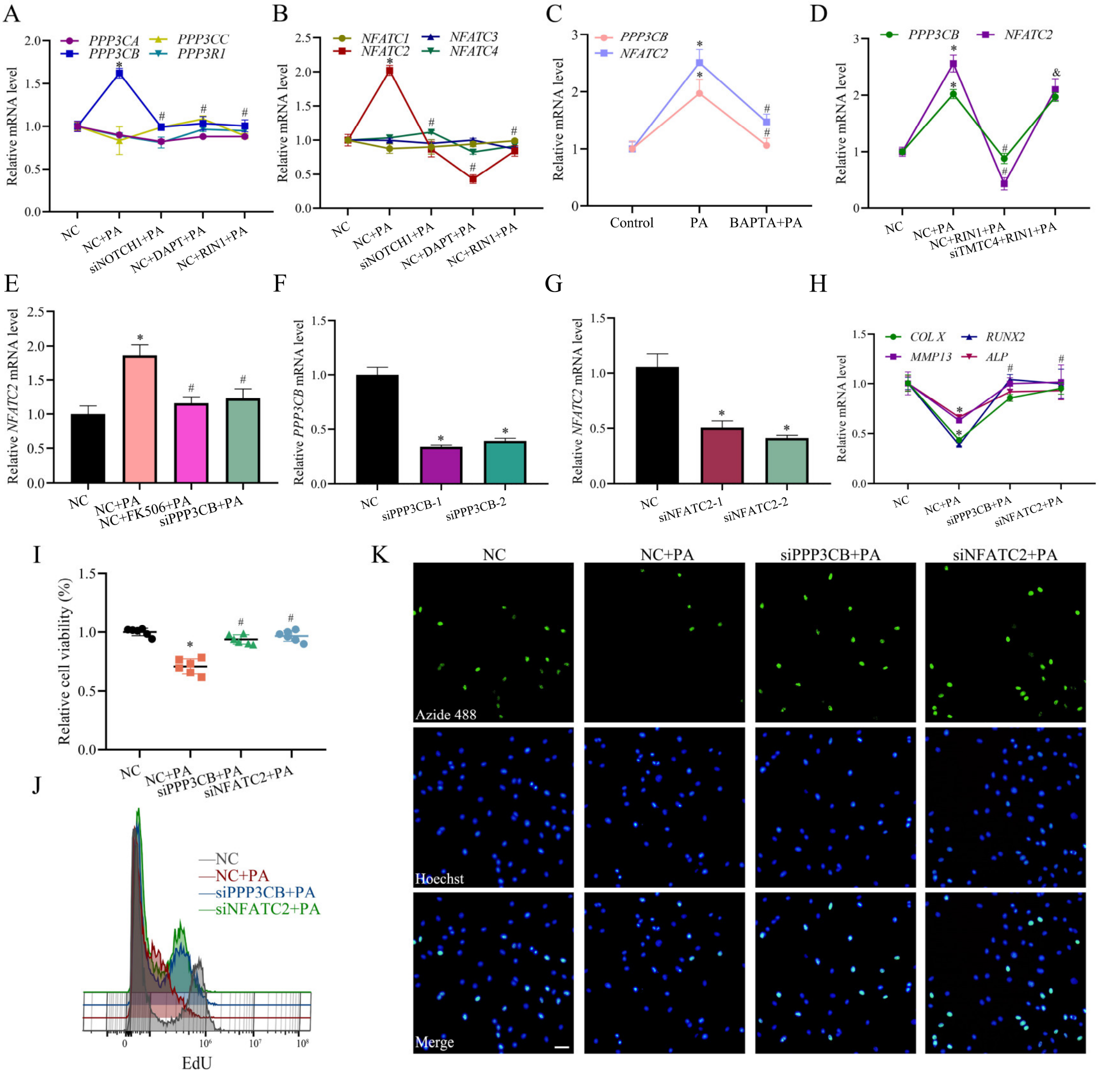

Figure S6. PA controlled the hypertrophy and proliferation of antler chondrocytes via Ca^{2+}-mediated PPP3CB-NFATC2 pathway. (A and B) Analysis of *PPP3CA*, *PPP3CB*, *PPP3CC*, *PPP3R1*, *NFATC1*, *NFATC2*, *NFATC3* and *NFATC4* mRNA expression after introduction of scrambled or NOTCH1 siRNA followed by the addition of PA or DAPT and RIN1 plus PA, respectively. *n*=3. **(C)** Regulation of PA on the expression of *PPP3CB* and *NFATC2* mRNA in the absence or presence of BAPTA. *n*=3. **(D)** Assessment of *PPP3CB* and *NFATC2* mRNA expression after introduction of scrambled or TMTC4 siRNA followed by the addition of PA or RIN1 plus PA. *n*=3. **(E)** Assessment of *NFATC2* mRNA expression after introduction of scrambled or PPP3CB siRNA followed by the addition of PA or FK506 plus PA. siPPP3CB, PPP3CB siRNA. *n*=3. **(F)** Measurement of *PPP3CB* mRNA expression after introduction of PPP3CB siRNAs. *n*=3. **(G)**

Determination of *NFATC2* mRNA expression after transfection with NFATC2 siRNAs. siNFATC2, NFATC2 siRNA. *n*=3. **(H)** Determination of *COL X*, *MMP13*, *RUNX2* and *ALP* expression after introduction of PPP3CB or NFATC2 siRNA followed by an addition of PA. *n*=3. **(I)** Detection of chondrocyte viability after the above treatment. *n*=6. **(J)** Flow cytometry analysis of chondrocyte proliferation behind above treatment. *n*=3. **(K)** Visualization of chondrocyte proliferation after the above treatment. Scale bar, 50 μm. *n*=3. * $P < 0.05$ versus NC or control, # $P < 0.05$ versus PA or NC plus PA treatment, & $P < 0.05$ versus NC and RIN1 plus PA treatment.

Figure S7

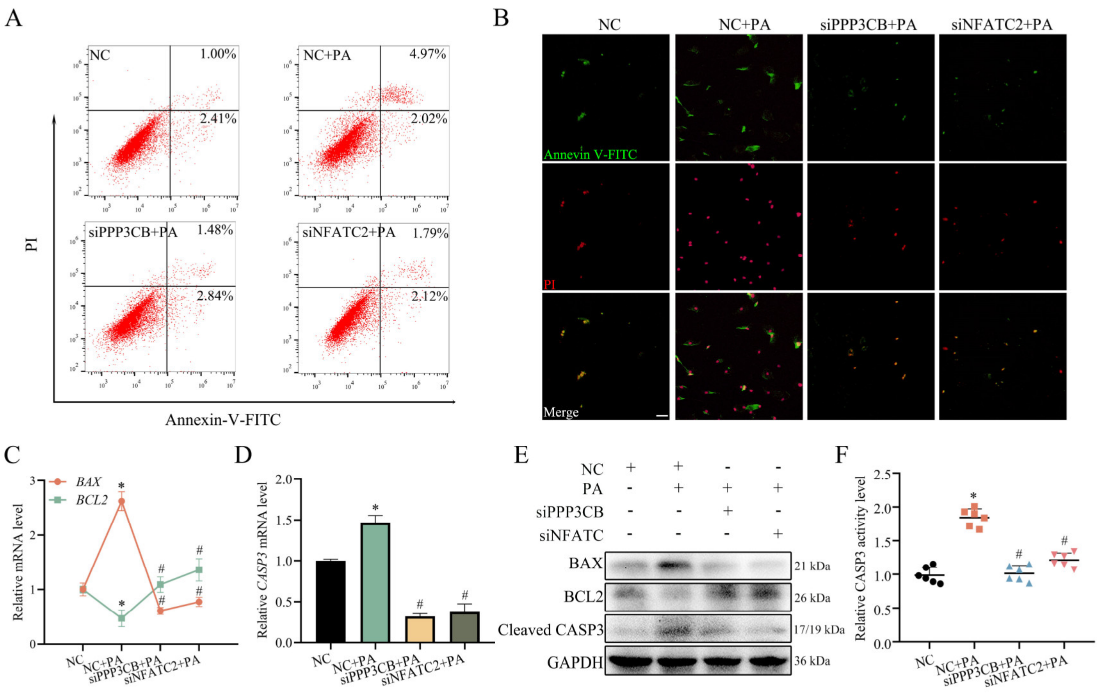

Figure S7. PA controlled the apoptosis of antler chondrocytes via PPP3CB-NFATC2 pathway. **(A)** Flow cytometry analysis of chondrocyte apoptosis after introduction of PPP3CB or NFATC2 siRNA followed by an addition of PA. *n*=3. **(B)** Visualization of chondrocyte apoptosis after the above treatment. Scale bar, 50 μm. *n*=3. **(C and D)** Determination of *BCL2*, *BAX* and *CASP3* mRNA expression after the above treatment. *n*=4. **(E)** Detection of BCL2, BAX and cleaved CASP3 protein expression after the above treatment. *n*=3. **(F)** Measurement of CASP3 activity after the above treatment. *n*=6. * $P < 0.05$ versus NC, # $P < 0.05$ versus NC plus PA treatment.

Figure S8

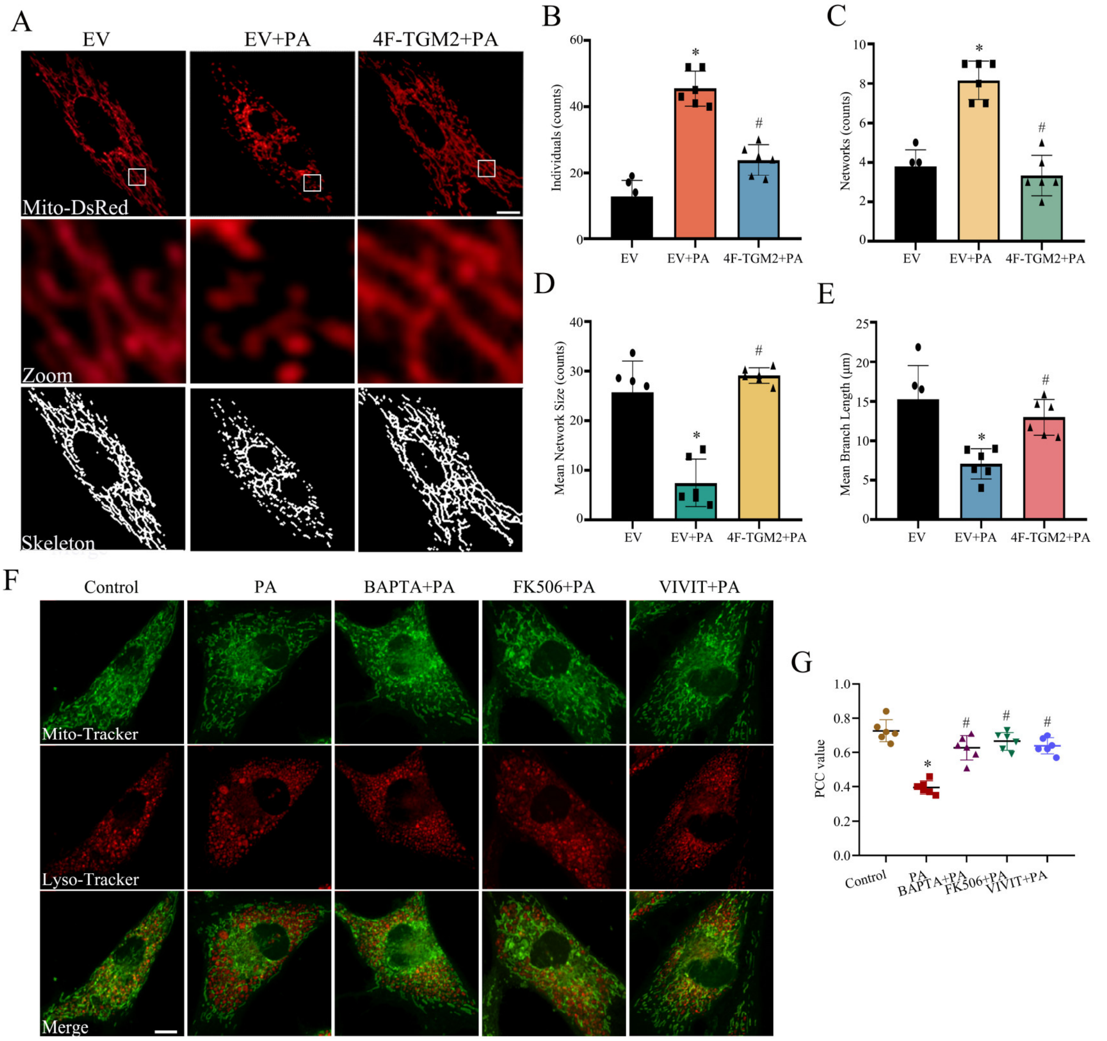

Figure S8. Effect of PA on mitochondrial morphology and mitophagy. (A) Visualization of mitochondrial network variation after introduction of mito-DsRed and TGM2 overexpression plasmids along with an addition of PA. The white box indicated the region magnified in the lower inset. Scale bar, 10 μm. **(B-E)** Analysis of mitochondrial network morphology using ImageJ software. *n*=6. **(F and G)** Visualization of mitochondria and lysosomes colocalization followed by the analysis of PCC value after treatment with PA in the absence or presence of BAPTA, FK506 and VIVIT. Scale bar, 10 μm. *n*=6. * $P < 0.05$ versus EV or control, $^{\#}P < 0.05$ versus PA or EV plus PA treatment.

Figure S9

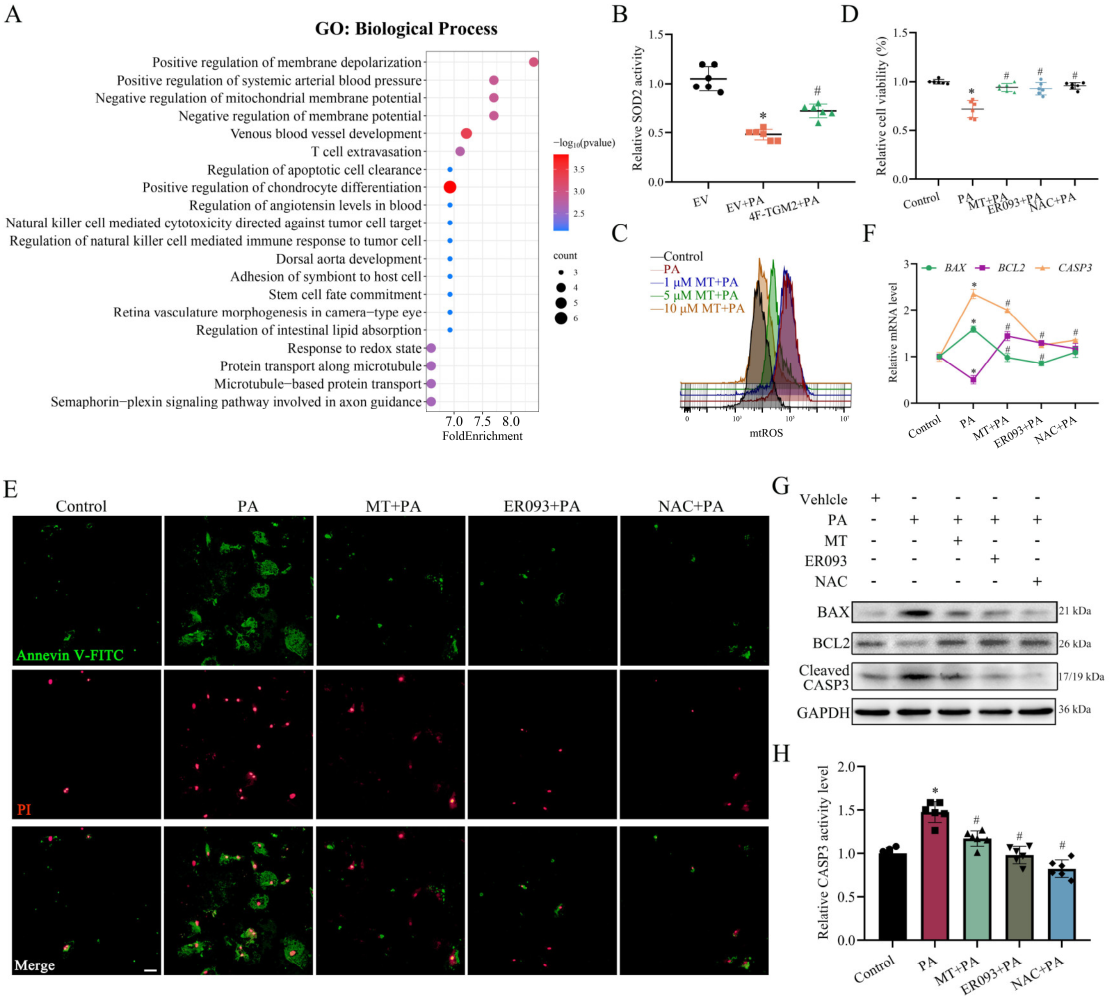

Figure S9. Effects of PA on the proliferation and apoptosis of antler chondrocytes via mPTP-mediated mtROS leakage. (A) GO analysis of HEGs in antler cartilage layers. **(B)** Measurement of SOD2 activity after introduction of TGM2 overexpression plasmid and addition of PA. *n*=6. **(C)** Flow cytometry analysis of mtROS level after treatment with different concentrations of mitochondria-targeted ROS scavenger MT under the context of PA. *n*=3. **(D)** Detection of chondrocyte viability after treatment with PA in the absence or presence of MT, mPTP opening inhibitor ER093 and ROS scavenger NAC. *n*=6. **(E)** Visualization of chondrocyte apoptosis behind above treatment. Scale bar, 50 μm. *n*=3. **(F)** Determination of *BCL2*, *BAX* and *CASP3* mRNA expression after the above treatment. *n*=3. **(G)** Detection of BCL2, BAX and cleaved CASP3 protein expression after the above treatment. *n*=3. **(H)** Measurement of CASP3 activity after the above treatment. *n*=6. * $P < 0.05$ versus control, # $P < 0.05$ versus PA treatment.

Table S1. Homologous siRNA sequences used in this study

siRNA Name	Target Gene	Sense Sequence	Accession number
siNOTCH1-1	NOTCH1	GCUGCGAGAUCGACGUCAATT	XM_043916357
siNOTCH1-2	NOTCH1	GGAGCAUGUGUAACAUCAATT	
siTMTC4-1	TMTC4	GGAGCUACGAUGGAGACUUTT	XM_043892475
siTMTC4-2	TMTC4	GGGCCAUAAACUAUAAUUATT	
siTMTC4-3	TMTC4	GCCUGGGAUUCCUCAUUAUTT	
siPPP3CB-1	PPP3CB	GGGUUGAUGUUCUGAAGAATT	XM_043924890
siPPP3CB-2	PPP3CB	GCUAUAGAAUGUACAGAAATT	
siNFATC2-1	NFATC2	AGAUGUUUGUUUACACUAATT	XM_043884593
siNFATC2-2	NFATC2	GCUGCUUGAUAAUAUAUAATT	
NC	N/A	UUCUCCGAACGUGUCACGUTT	

Table S2. Primer sequences using for qPCR

Gene	Sequence of Forward Primer	Sequence of Reverse Primer	Accession number
NOTCH1	AACCGTAGCTCCTGAGAGCA	AGAGTCTGATCGTGCCCACT	XM_043916357
NOTCH2	TCGCTTCCAGTGTCTGTGTC	ACACTTTGCCCCATTCAGAC	XM_043877037
RBPJ	GAGCGAGGGGATCAAACAGT	CAGAACAACCATCGCGTTCC	XM_043871438
BAX	CGGGTTGTCGCCCTTTTCTA	CGCTCTCGAAGGAAGTCCAA	XM_043899754
BCL2	GAGCAGCTCTAACTGGAGAGT	CTACCTCCTCCGTGATGTTGT	XM_043889675
CASP3	TGGAACAAATGGACCCGTCA	ACCACTGTCTGTCTCGATGC	XM_043893681
COL X	ATCCCCGGCCCAGCTGGAAT	GGGAGGCCCCTCTCACCTGG	XM_043889918
RUNX2	TCAGAACCCACGGCCCTCCC	GACAGCGGCGTGGTGGAGTG	XM_043907627
MMP13	CCTCTGGTCTGTTGGCTCACGC	GCTCCTGGGTCCTTGGAGTGGT	XM_043893458
ALP	GGAAGGGGGCAGGATTGAC	GGTGTACCCGCCAAAGGTAA	XM_043909115
HES1	AGAGGCGGCTAAGGTGTTTG	CATGGAGTCTGCCGTGAGAG	XM_043875191
HES5	ACTTCTGGGAAGTCCCCACT	CCGCCTTCACAAAAGGCAAA	XM_043923957
HES7	CGAAGCCGGGTGGAGC	CGGAATCCCGACAAGTAGCA	XM_043900932
HEY1	GTTCGCCTGGTCTCACATCT	CTGGGACAGTAGTAGGGGGT	XM_043880250
HEY2	CGAGAACAATTACTCGGGGCA	GGTTGGCACCAGTCGTCTC	XM_043890285
HEYL	CGTCGGATACACAGCACAGA	GACTGTCCTTCTTCTCGCCC	XM_043877232
TMTC4	ATGCTGGTCAGGGCCATAAA	TGCAACCCATCGACCAATCA	XM_043892474
SERCA2	TTGCTCGAGTTGAACCCTCC	AGAAGTTGTCATCCGCCAGG	XM_043901877
PPP3CA	GGGGCTTCCATTCTTCGACA	AAGAGGTAGCGAGTGTTGGC	XM_043871132
PPP3CB	CCCCAACACATCGCTTGACAT	GGCAGCACCCTCATTGATAATTC	XM_043924886
PPP3CC	ACCGCGTCATCAAAGCTGT	CTTCCAGTCGTCCTTCCTTTAC	XM_043927276
PPP3R1	CCTTTGGAAATGTGCTCACACT	GGATTCTGTTGTAACTCAGGCAG	XM_043917614
NFATC1	GCCATCCTCTCCAACACCAA	ACCCGTGTGTTCTTTCTCCC	XM_043889600
NFATC2	GAGGCCACGGTGGATAAAGA	CGGGACTGGGTGGTAGGTAA	XM_043884589
NFATC3	AGACACCTGGCTCAATGCTC	TTTCCCGCGATGGTGAAAGA	XM_043898875
NFATC4	TCCACTGGCTTGGTCCAAGG	AGCTGCTCATACTGGCTGGG	XM_043920303
TGM2	CGAGTTCGGGGAGATCCAGA	CACAGCAGTAAGTCCCTTCGC	XM_043883725
GAPDH	GAAGGGTGGCGCCAAGAGGG	GGGGGCCAAGCAGTTGGTGG	XM_043881239

Table S3. Primary antibodies for western blotting

Antibodies name	Source	**Identifier**
N1ICD sheep polyclonal antibody	R&D Systems	AF3647
RBPJ rabbit polyclonal antibody	Proteintech	14613-1-AP
BCL2 rabbit polyclonal antibody	Abmart	T40056
BAX rabbit polyclonal antibody	Proteintech	50599-2-Ig
Cleaved CASP 3 rabbit polyclonal antibody	Cell Signaling Technology	9661
FLAG-tag mouse mAb	ABClonal	AE005
GAPDH rabbit polyclonal antibody	Proteintech	60004-1-Ig
Histone H3 rabbit polyclonal antibody	Proteintech	17168-1-AP
